# Evaluation of Anthelmintic and Anti-Inflammatory Activity of 1,2,4-Triazole Derivatives

**DOI:** 10.3390/molecules27144488

**Published:** 2022-07-13

**Authors:** Renata Paprocka, Przemysław Kołodziej, Małgorzata Wiese-Szadkowska, Anna Helmin-Basa, Anna Bogucka-Kocka

**Affiliations:** 1 Department of Organic Chemistry, Faculty of Pharmacy, Collegium Medicum in Bydgoszcz, Nicolaus Copernicus University in Toruń, Jurasza Str. 2, 85-089 Bydgoszcz, Poland; 2 Chair and Department of Biology and Genetics, Faculty of Pharmacy, Medical University in Lublin, Chodźki Str. 4A, 20-093 Lublin, Poland; anna.bogucka-kocka@umlub.pl; 3 Department of Immunology, Faculty of Pharmacy, Collegium Medicum in Bydgoszcz, Nicolaus Copernicus University in Toruń, M. Curie-Sklodowska Str. 9, 85-094 Bydgoszcz, Poland; mwiese@cm.umk.pl (M.W.-S.); a.helmin-basa@cm.umk.pl (A.H.-B.)

**Keywords:** parasitic diseases, *Rhabditis* sp., nematodes, nematicidal activity, nematicides, amidrazone, PBMC, antiproliferative activity, antiparasitic activity

## Abstract

Parasitic diseases, caused by intestinal helminths, remain a very serious problem in both human and veterinary medicine. While searching for new nematicides we examined a series of 1,2,4-triazole derivatives **9**–**22,** obtained during reactions of *N*^3^-substituted amidrazones with itaconic anhydride. Two groups of compounds, **9**–**16** and **17**–**22,** differed in the position of the double bond on the methacrylic acid moiety. The toxicity of derivatives **9**–**22** and the anti-inflammatory activity of **12** and **19**–**22** were studied on peripheral blood mononuclear cells (PBMC). Antiproliferative activity of compounds **12** and **19**–**22** was tested cytometrically in PBMC cultures stimulated by phytohemagglutinin. The influence of derivatives **12** and **19**–**22** on the TNF-α, IL-6, IL-10 and IFN-γ production was determined by ELISA in lipopolysaccharide-stimulated PBMC cultures. Anthelmintic activity of compounds **10**–**22** was studied in the *Rhabditis* sp. nematodes model. Most compounds (**11**–**22**) proved to be non-toxic to human PBMC. Derivatives **19**–**22** showed anti-inflammatory activity by inhibiting the proliferation of lymphocytes. Moreover, compounds **12** and **19**–**22** significantly reduced the production of TNF-α and derivatives **19**–**21** decreased the level of INF-γ. The strongest anti-inflammatory activity was observed for compound **21**. Compounds **12** and **14** demonstrated anthelmintic activity higher than albendazole and may become promising candidates for anthelmintic drugs.

## 1. Introduction

Parasitic diseases, including intestinal parasitic infections, are caused by various species of parasites, and constitute a serious health and socioeconomic problem. Globally, it is estimated that infections caused by intestinal parasites affect 3.5 billion people, 450 million of whom are symptomatic and have health problems as a direct result of the disease. Nematodes are largely responsible for these diseases, including *Ascaris lumbricoides*, *Trichiuris trichiuria*, *Ancylostoma duodenale* and *Strongyloides stercoralis* [1,2,3,4,5,6]. Diseases caused by intestinal nematodes can cause, among others, abdominal pain, dehydration, weight loss, anemia, intestinal obstruction, disorders of mental and physical development, malabsorption, inflammation and in severe cases, if left untreated, these can even lead to death [6,7,8].

Currently, a narrow group of drugs is used in the treatment of parasitic diseases caused by helminths, mainly albendazole, mebendazole and semi-synthetic ivermectin. Anthelmintic agents are characterized by a variety of structures and mechanisms of action, which makes it difficult to develop a simple route in the search for new drugs [9,10,11,12,13,14,15,16]. Although there is an urgent need for new antiparasitic drugs, including anthelmintics, a new generation of drugs is still not available. This lack of investment in research is mainly caused by the fact that there is no interest from the pharmaceutical industry, as most parasitic diseases occur in poor countries and there is a high risk of no return on investment [17]. However, an increase in helminths’ resistance to available drugs has been observed in recent years [18,19,20]. Therefore, it is important to conduct scientific research using both natural and synthetic chemical compounds to obtain new and effective anthelmintic drugs. Many plants have drawn attention as a potential source of anthelmintic substances [9,10,21,22]. The anthelmintic activity of synthetic chemical compounds has also been demonstrated, including that of thiosemicarbazide derivatives [23,24]. Our research is in line with the trend moving towards discovering new synthetic anthelmintic compounds.

Currently, research concerning the bioactivity of triazole derivatives is very popular. Many different biological activities of 1,2,4-triazole derivatives have been described in the literature, e.g., antibacterial, antifungal, antiviral and antitumor [25,26,27,28,29]. However, there are very few reports on the anthelmintic activity of triazole derivatives and these are mainly studies conducted on earthworms, not nematodes specifically [30,31,32].

Nematodes of the genus *Rhabditis* sp. are free-living organisms found mainly in organic debris and soil [33]. However, there have been reports in the literature that this nematode can cause infections in humans, e.g., of the urinary tract, outer ear canal and digestive system [33,34,35,36,37,38,39,40].

In our previous publication, we described some 1,2,4-triazole derivatives which possessed methacrylic acid moieties, promising anti-inflammatory activity and low toxicity towards human lymphocytes [41]. Continuing our study on amidrazone derivatives, we took up the idea that compounds possessing a 1,2,4-triazole ring similar to (but containing one extra nitrogen atom) the diazole moiety present in anthelmintic drugs like albendazole or mebendazole could also be active against nematodes. The aim of this study was to determine the anthelmintic and anti-inflammatory activities of newly synthesized and already known 1,2,4-triazole derivatives and the anti-inflammatory activity of the new compounds.

## 2. Results

### 2.1. The Synthesis of ***9***–***22***

**Scheme 1:**
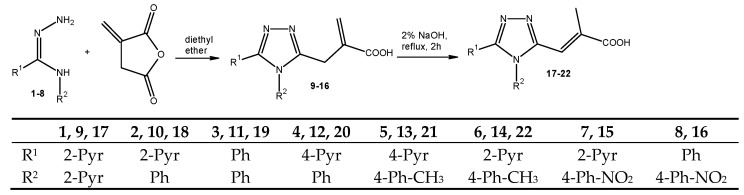
The synthesis of compounds **9**–**22**.

1,2,4-triazole derivatives **9**–**16** were obtained following previously established procedures via the reaction of amidrazones **1– 8** [42] with itaconic anhydride [41]. Consequently, isomerization of compounds **9**–**14** was undertaken in alkalic solution, leading to derivatives **17**–**22** (Scheme 1). Considerably higher yields of isomerization were obtained for compounds **20** and **21**, which may suggest that a 4-pyridine ring, found in the R^1^ position, facilitates this reaction. In contrast, no isomerization products were obtained following the reactions of 1,2,4-triazole derivatives **15** and **16**, which may suggest that the presence of a 4-nitrophenyl substituent hinders this reaction. Structures of new compounds were confirmed by ^1^H NMR, ^13^C NMR and MS and their purities by elemental analyses.

The main structural difference between derivatives **9**–**14** and **17**–**22** was the presence of a methylidene CH_2_= moiety attached to propanoic acid in compounds **9**–**14** and the presence of a methyl (CH_3_) group attached to (*E*)-propenoic acid in compounds **17**–**22**. Derivatives **17**–**22** were also more thermodynamically stable than compounds **9**–**14** which is beneficial for pharmacological use. The influence of this structural difference on anthelmintic and anti-inflammatory activity was the subject of further biological research.

### 2.2. Biological Assays In Vitro

Human PBMC represented a tissue source in the biological part of the presented studies. Interestingly, PBMC are widely used as a physiological model for immunological research of parasitic infections, such as those caused by *Toxoplasma gondii* and others [43]. PBMC are routinely isolated from blood samples (as we describe previously in [44]) and then used in several different tests to determine the toxicity, anti-proliferative activity and anti-inflammatory activity of studied compounds. We used the cytometric method to evaluate the proliferation response to phytohemagglutinin (PHA) and immunoezymatic tests to verify the levels of proinflammatory cytokines after lipopolysaccharide (LPS) stimulation. The cytokines from the early immune response that were tested include: TNF-α, IL-6 and IL-10. Additionally, we verified the level of cytokines from adaptive immunity—namely, IFN-γ. IFN-γ is a very important cytokine which takes part in immune responses against parasites [45].

#### 2.2.1. Toxicity of Compounds **9**–**22**

Most of the studied 1,2,4-triazole derivatives **11**–**22** were characterized by low toxicity towards PBMC at a concentration range up to 100 µg/mL (the range of toxicity at the highest compound concentration was 6.60–28.96% of apoptotic and necrotic cells, Figure S21, Supplementary Materials). Only derivatives **9** and **10** possessed a higher toxicity (only 52.7 and 43.40% of viable cells in 24 h PBMC culture, respectively). To compare the results, we also prepared the PBMC culture with a nonsteroidal anti-inflammatory drug—ibuprofen (the reference control); the toxicity level was 29.53% of apoptotic and necrotic cells [41]. It should be noted that all compounds that had a higher toxicity level than 30% were excluded from our research. In our previous study, PBMC cultures treated with compounds **9**–**11** and **13**–**18** showed 71.04–82.39% viable cells [41]. The percentage of viable cells for new compounds **12**, **19**–**22** was in the range of 90.56–93.40%, which is higher than the control culture containing the same concentration of DMSO (87.27%). For comparison, five by six 1*H*-pyrrole-2,5-dione derivatives (obtained from the same amidrazones **1**–**8** as compounds **11**–**22**) showed about 79–92% of viable cells [46]. In general, derivatives **17**–**22** were less toxic than **9**–**16** while compounds **10** and **18** possessing phenyl and 2-pyridine rings were the most toxic within those two groups.

#### 2.2.2. Antiproliferative Activity of **12** and **19**–**22**

**Figure 1:**
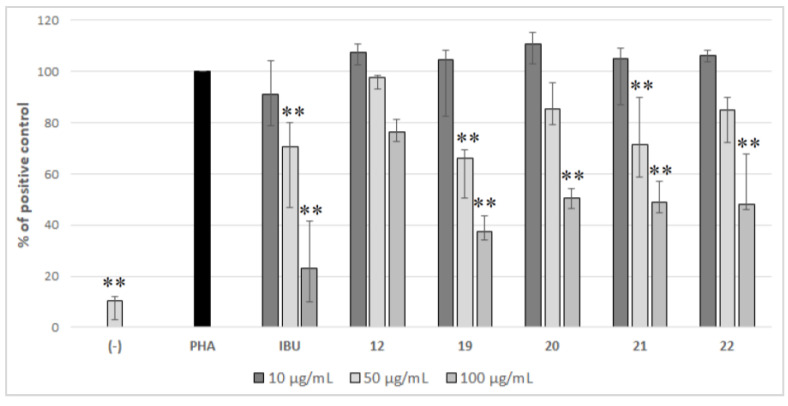
The effect of compounds **12**, **19**–**22** and IBU on the PHA-induced PBMC proliferation in 72 h cell cultures (the results are shown as percentage of positive control (PHA alone), with values expressed as medians from six independent experiments and interquartile ranges [Q1, Q3]); ** indicates a significant difference compared to positive control (PHA alone) at *p* < 0.01.

Flow cytometry assay was used to find the cytotoxic potential of compounds upon proliferation of PHA-induced PBMC. Compounds **19**, **21** and IBU (reference drug) were inhibitory at a concentration of 50 µg/mL. All compounds (**12**, **19**–**22**) as well as IBU inhibited the proliferation of PBMC at a concentration of 100 µg/mL (the strongest inhibitory effect was observed for IBU and compound **19** causing 77.0% and 62.4% of inhibition, respectively; Figure 1). Significant inhibition of PBMC proliferation was also observed for previously studied compounds **13** and **17**–**18** (nearly 100% at a dose of 100 µg/mL and 80% at 50 µg/mL) [41]. None of the compounds (**12** and **19**–**22**) inhibited PBMC proliferation at the lowest concentration of 10 µg/mL.

#### 2.2.3. The Influence of Compounds **12** and **19**–**22** on Cytokine Production

The influence of newly synthetized compounds and IBU was studied on a PBMC model. PBMC were induced by LPS (an endotoxin from gram-negative bacteria used to elicit an inflammatory response in vitro). Stimulating PBMC with LPS produces a high level of pro-inflammatory cytokines such as TNF-α, IL-6, IFN-γ and anti-inflammatory cytokines IL-10 [47].

#### 2.2.4. TNF-α

**Figure 2:**
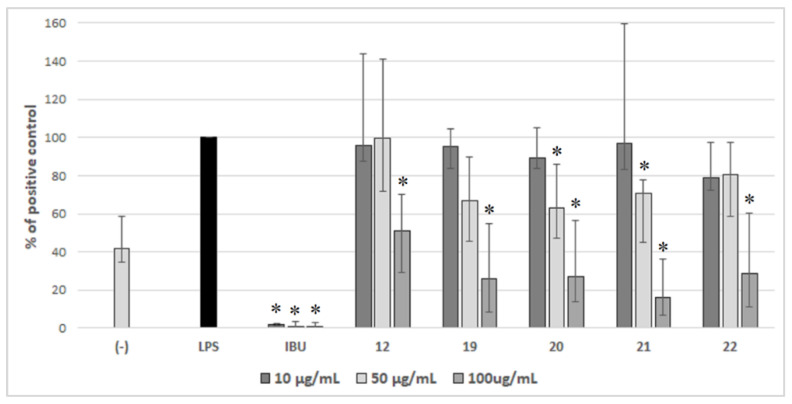
The effect of compounds **12**, **19**–**22** and IBU on the LPS-induced production of TNF-α in PBMC cultures (the results are shown as percentage of positive control (LPS alone), with values expressed as medians from previous independent experiments and confidence intervals); * indicates a significant difference compared to the positive control (LPS alone) at *p* < 0.05.

IBU significantly reduced the level of TNF-α in PBMC stimulated by LPS at all used doses (by about 99%). Compounds **12** and **19**–**22** significantly inhibited the production of TNF-α only at a concentration of 100 µg/mL (Figure 2). The strongest activity was observed for **21** (about 84% of inhibition), while compounds **19**, **20** and **22** caused about 71–74% of inhibition. Inhibitory effects were also observed for derivatives **20**–**21** at concentrations of 50 µg/mL. Compound **12** inhibited the production of TNF-α only at the highest dose (about 50% of inhibition). Derivatives **13** and **17**–**18** revealed stronger suppression of TNF-α production, especially in low doses (nearly 100% at 10 µg/mL, about 75% at 50 µg/mL and about 50% at 100 µg/mL) [41].

#### 2.2.5. IL-6

**Figure 3:**
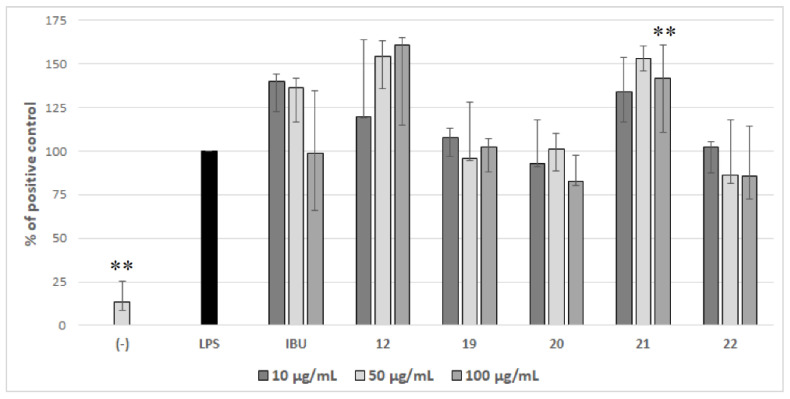
The effect of compounds **12**, **19**–**22** and IBU on the LPS-induced production of IL-6 in PBMC cultures (the results are shown as percentage of positive control (LPS alone), with values expressed as medians from five independent experiments and confidence intervals); ** indicates a significant difference compared to positive control (LPS alone) at *p* < 0.01.

Compounds **12** and **19**–**22** had no significant effect on IL-6 production with the exception of compound **21**, which elevated IL-6 production at a concentration of 100 µg/mL (Figure 3). This result is in agreement with our previous studies—compounds **9**–**11** and **13**–**18** demonstrated no significant influence on IL-6 production [41].

#### 2.2.6. IL-10

**Figure 4:**
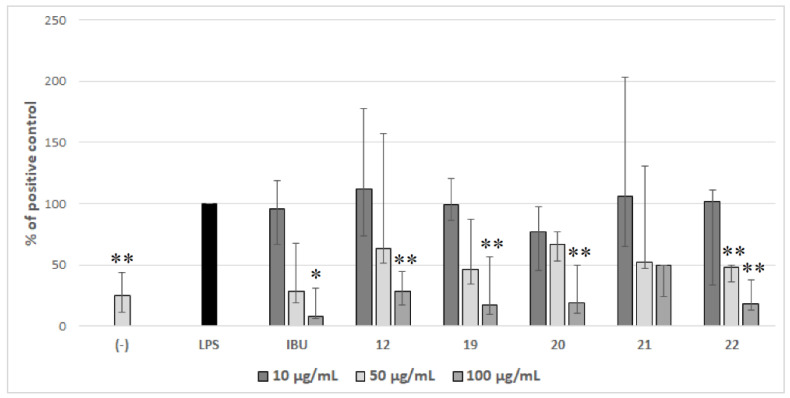
The effect of compounds **12**, **19**–**22** and IBU on the LPS-induced production of IL-10 in PBMC cultures (the results are shown as percentage of positive control (LPS alone), with values expressed as medians from five independent experiments and confidence intervals); * indicates significant difference comparing to positive control (LPS alone) at 0.01 < *p* < 0.05; ** indicates a significant difference compared to positive control at *p* < 0.01.

In the case of LPS-induced IL-10 production, neither of the compounds significantly increased the release of this cytokine. However, an inhibition of IL-10 production was observed at a concentration of 100 µg/mL for **12**, **19**, **20**, **22** (about 71–83% inhibition) and IBU (92.3% inhibition) as well as for **22** at a concentration of 50 µg/mL (Figure 4). It is noteworthy that compound **21** inhibited the production of IL-10 the least.

#### 2.2.7. INF-γ

**Figure 5:**
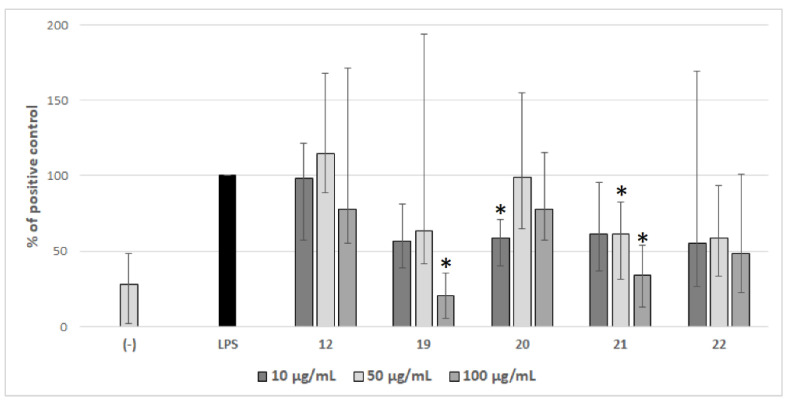
The effect of **12** and **19**–**22** on the LPS-induced production of INF-γ in PBMC cultures (the results are shown as percentage of positive control (LPS alone), with values expressed as medians from previous independent experiments and confidence intervals); * indicates a significant difference compared to positive control (LPS alone) at *p* < 0.05.

Newly synthetized compound **19** (high dose), **20** (low dose) and **21** (middle and high dose) significantly reduced the production of IFN-γ (Figure 5). Interestingly, using a similar model, researchers have shown that this factor is very important for the transmission of some parasites, because it increases the expression of an adhesion marker such as intercellular adhesion molecule-1 (ICAM-1) [48]. Thus, it can be concluded that the reduction of IFN-γ production is beneficial in counteracting parasitic infections.

#### 2.2.8. Anthelmintic Activity of Compounds **10**–**22**

**Figure 6:**
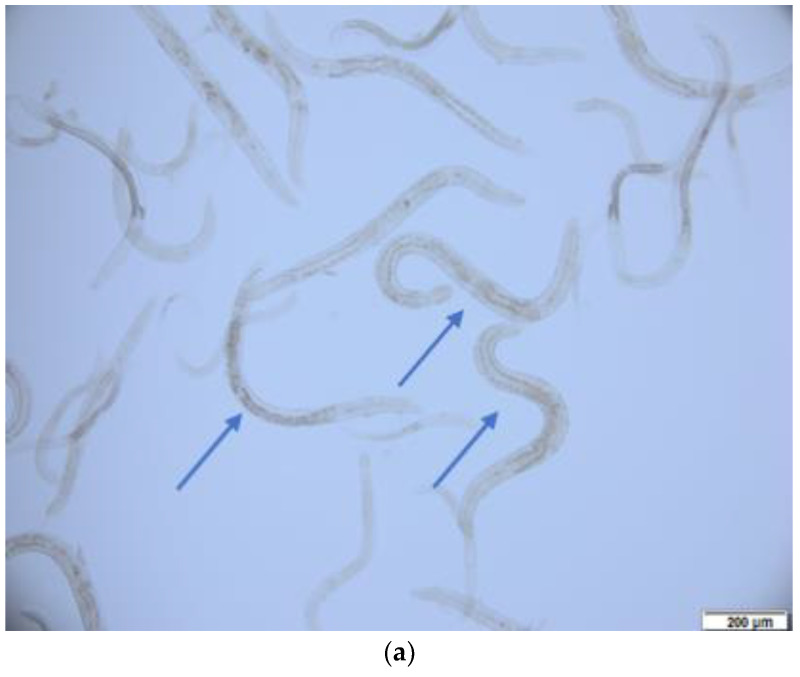

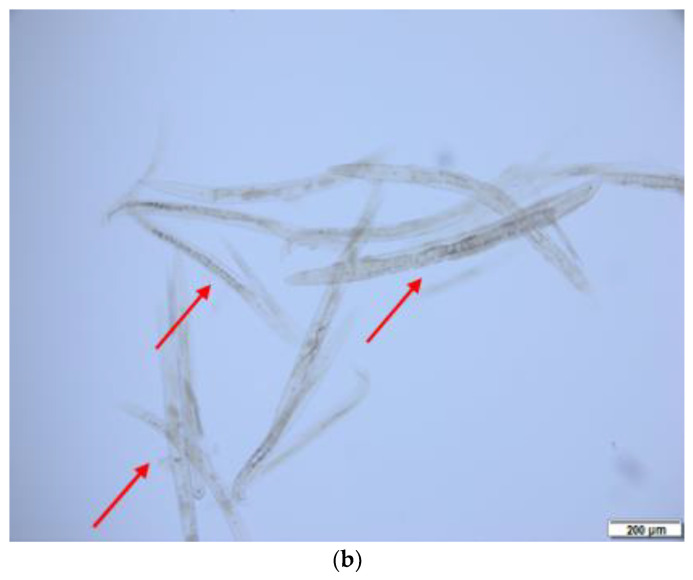
An exemplary nematode culture of *Rhabditis* sp. after 24 h exposure to compound **12**. (**a**) control (blue arrows—alive, mobile), (**b**) after treatment (red arrows—dead, non-mobiles).

**Figure 7:**
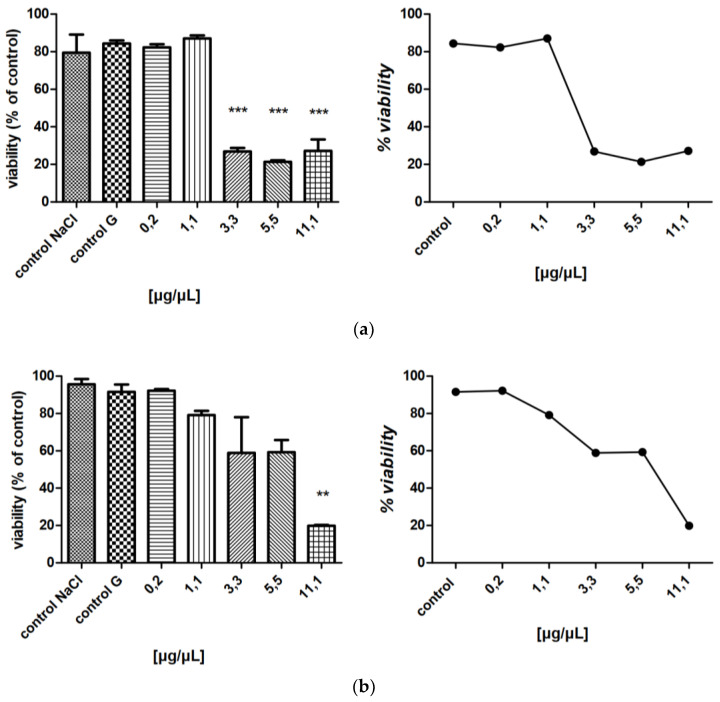

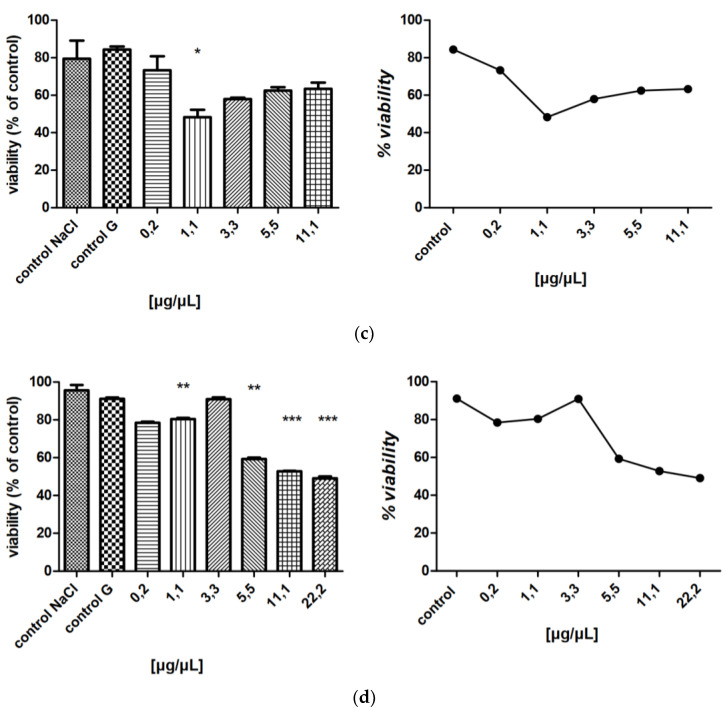
Viability (%) of *Rhabditis* sp. nematodes after 24 h exposure to increasing concentrations of the compounds (**a**) **12,** (**b**) **14,** (**c**) **18** and (**d**) albendazole [38,49] (*p* < 0.05 *, *p* < 0.01 **, *p* < 0.001 ***).

Compound **12,** which possesses 4-pyridyl and phenyl substituents (at R^1^ and R^2^ positions, respectively), demonstrated the highest anthelmintic activity with a lethal concentration LC_50_ (the amount of an ingested substance that kills 50 percent of a test sample) of 2.475 ± 0.283 µg/µL (Figure 6 and Figure 7). Moreover, compound **14**, bearing 2-pyridyl and 4-methylphenyl substituents, was characterized by anthelmintic activity (LC_50_ = 6.550 ± 0.866 µg/µL). Both compounds were more effective than albendazole (LC_50_ = 19.24 µg/µL) [49]. Derivative **18** possessed significant anthelmintic activity only at a dose of 1.1 µg/µL (Figure 7). Compounds **10**, **11**, **13**, **15**–**17** and **19**–**22** were characterized by a lack of anthelmintic activity.

## 3. Discussion

Continuing our previous research on 1,2,4-triazole derivatives obtained during the reaction between *N*^3^-substituted amidrazones and itaconic anhydride [41], we examined two groups of compounds, **9**–**16** and **17**–**22**, differing in the position of the double bond in the side chain of methylacrylic acid. Anthelmintic and anti-inflammatory activities of obtained compounds were studied to determine the influence of their structure on their biological properties.

Derivatives **11**–**22** were not toxic to human PBMC at a concentration of 100 µg/mL in a physiological model. In our previous study, derivatives **13** and **17**–**18** revealed the strongest anti-inflammatory activity among compounds **11** and **13**–**18** by inhibiting both TNF-α production and proliferation of PBMC [41]. As a continuation of this work, we extended the research on the anti-inflammatory activity of derivatives **12** and **19**–**22**. Compounds **12** and **19**–**22** (at concentration of 100 µg/mL) and derivatives **20**–**21** (50 µg/mL) significantly inhibited production of TNF-α in LPS-stimulated PBMC (but were less effective than the IBU, Figure 2). Neither of the studied compounds lowered the level of IL-6 (Figure 3). All compounds (**12** and **19**–**22**, except for **21**) showed a similar inhibitory effect on the synthesis of anti-inflammatory IL-10 as IBU (Figure 4). Compounds **19**–**22** (at a concentration of 100 µg/mL) and derivatives **19** and **21** (50 µg/mL) significantly inhibited lymphocyte proliferation in a similar way to reference IBU (Figure 1). Comparing the results with earlier studies, we confirmed that derivatives **17**–**22** (which possess a methyl group in propenoic acid moiety) have stronger antiproliferative properties in PHA-induced PBMC cultures than compounds **11**–**16** (which contain a CH_2_= group in a propanoic acid moiety) [41].

We suspected that compound **21**, which contains the same 4-pyridyl and 4-methyphenyl substituents as **13** (the strongest anti-inflammatory agent among **11** and **13**–**16**), and the favorable position of the double bond will be the most active derivative in the current study. In fact, compound **21** was the only one among derivatives **19**–**22** which inhibited lymphocyte proliferation and the production of pro-inflammatory cytokines (TNF-α and IFN-γ) at two doses: 50 µg/mL and 100 µg/mL in mitogen-stimulated PBMC cultures. Moreover, derivative **21** was the only one among compounds **17**–**22** that did not significantly decrease the production of the anti-inflammatory cytokine IL-10 at all studied concentrations. Summing up, compound **21** has shown the most promising properties among derivatives **19**–**22** and it is worth future research as anti-inflammatory agent.

Afterwards, we studied the anthelmintic potential of compounds **11**–**22** on *Rhabditis* sp. nematodes. Our research model was similar to the *Caenorhabditis elegans* often used in research [50]. *Rhabditis* sp. nematodes were used, among others, to determine the anthelmintic activity of the new rhodanine derivatives of cinnamaldehyde [49], naphthalimide—conjugates of boron clusters [51]—and thiosemicarbazide derivatives [24].

According to our knowledge, there are only few reports in the literature on the anthelmintic activity of 1,2,4-triazole derivatives. In previous research on Indian worm *Pheritima postuma* activity of 1,2,4-triazole, the strongest anthelmintic activity showed derivatives possessing 2,4-dichlorophenyl [52], 4-chlorophenyl or 4-nitrophenyl moieties [53]. The diversity of substituents present in the most active compounds indicates various possibilities for the modification of potential nematicidal agents.

Among 1,2,4-triazole derivatives **11**–**22** we studied in the *Rhabditis* model, compound **12** showed about eight times greater nematocidal activity than albendazole and compound **14** was approximately three times more active than the reference drug. When analyzing the results of the anthelmintic activity, we have also been able to detect a relationship between the structure and antiparasitic potential of studied compounds. The derivatives which possess a methylidene group CH_2_=, like **12** and **14**, showed stronger anthelmintic activity than compounds obtained by their isomerization (**20** and **22**). Among the second group of 1,2,4-triazole derivatives (**17**–**22**), only one compound **18** at a single dose 1.1 µg/µL possessed significant anthelmintic activity (Figure 7c), which indicates a lower nematocidal potential of derivatives **17**–**22**. We observed that compounds possessing a 4-nitrophenyl (**15** and **16**) or 2-pyridine moiety at the R^2^ position (**17**) were devoid of anthelmintic activity. However, the phenyl substituent at the R^2^ position could be connected with the anthelmintic activity of compounds **12** and **18**. The influence of the substituent at the R^1^ position on anthelmintic activity is not so clear, however, the presence of a 2- or 4-pyridyl substituent at the R^1^ position in conjunction with the phenyl or 4-methylphenyl substituent at R^2^ seems to be important.

In our previous study, compound **14** revealed low acute toxicity in mice (LC_50_ value of 1000 mg/kg i.p.), lack of neurotoxic activity and weak effects on the central nervous system (CNS) of mice [25]. It may suggest favorable pharmacological properties of a whole group of related 1,2,4-triazole derivatives (**11**–**22**). Moreover, 1,2,4-triazole derivatives **9**–**11** and **13**–**18** demonstrated moderate antimicrobial activity (MIC ≥ 100 µg/mL) [25]. This may suggest neutrality to the human intestinal flora of studied compounds **9**–**22**.

It’s worth noting that the 1,2,4-triazole ring present in studied compounds **11**–**22** may interact with the same molecular target as albendazole (colchicine binding side). Our reference anthelmintic drug has three nitrogen atoms in a similar configuration to the compounds we studied. The structural advantage of compound **12** above other compounds could be the 4-pyridyl substituent (R^1^), which would detach with a similar purpose to the sulfur atom present in albendazole. The phenyl substituent in the R^2^ position provides better lipophilicity than the compounds **9**, **15**–**17** having an additional nitrogen atom or nitro group, none of which showed nematocidal activity. Another important element of the structure of derivative **12** seems to be the presence of the CH_2_= group in a methacrylic acid moiety also present in the second nematocidal compound **14**.

A limited number of publications on the anthelmintic properties of triazoles indicate that further research is required. The next scientific step will be to conduct research in experimental in vivo models and to determine the molecular mechanism of action of 1,2,4-triazole derivatives **12** and **14**.

## 4. Materials and Methods

All reagents and solvents were acquired from Sigma-Aldrich (Burlington, MA, USA) or Avantor Performance Materials Poland S.A (Gliwice, Poland). ^1^H NMR and ^13^C NMR spectra were recorded on Bruker Avance spectrophotometers (300, 400 or 700 MHz) in DMSO-d6 using TMS as an internal standard. Elemental analyses were performed on a Vario MACRO CHN analyzer (ELEMENTAR Analysensysteme GmbH, Langenselbold, Germany). Melting points were determined on a Mel TEMP 1002D apparatus and are given uncorrected. All reactions were controlled by TLC chromatography.

### 4.1. General Methods of Synthesis

Compounds **9**–**22** were synthesized according to established general procedures [41]:

Amidrazones **1**–**8** were obtained from corresponding thioamides and 80% aqueous hydrazine hydrate solution [42]. 0.01 mol of amidrazones **1**–**8** and 0.01 mol (1.12 g) of itaconic anhydride were dissolved in 100 mL of anhydrous diethyl ether and left in room temperature for 7 days. Obtained crude solids **9**–**16** were filtered off and purified by crystallization from a methanol−water mixture (1:1) [41,54]. Compounds **17**–**22** were obtained by heating 0.25 g of derivatives **9**–**14** at boiling point with 20 mL of 2% NaOH aqueous solution for 2 h. After cooling down, the reaction mixture was filtered off. A 1% hydrochloric acid solution was added dropwise to the obtained filtrate for as long as precipitation was observed. Obtained crude solids of **17**–**22** were filtered off and purified by crystallization from water or from water with the addition of methanol [41,54]. The characteristics of the new compounds **12** and **19**–**22** are available in the Supplementary Materials (^1^H NMR and ^13^C NMR spectral data on Figures S1–S10, HRMS data on Figures S11–S15, HPLC data on Figures S16–S20). Compounds **9**–**11** and **13**–**18** were characterized in our previous publications [41,54,55].

### 4.2. Biological Assays In Vitro

#### 4.2.1. Peripheral Blood Mononuclear Cells Preparation

Peripheral blood mononuclear cells (PBMC) were isolated immediately from fresh blood as previously described [43]. After isolation, PBMC were used to conduct experiments which assessed toxicity of compounds and their impact on proliferation and cytokine production.

The cell suspensions (1 × 10^6^ cells/mL) in culture medium (5% fetal bovine serum—FBS—Euroclone SpA, Pero, Ml, Italy, in RPMI 1640—Biomed Lublin, Lublin, Poland) were added to 5 mL tubes (Falcon^®^ Round Bottom Polystyrene Tubes, Corning, NY, USA) in all experiments. Compounds **9**–**22** and ibuprofen (IBU) were initially dissolved in dimethyl sulfoxide (DMSO, Sigma-Aldrich) and afterwards, were added in appropriate amounts to the cell culture. Final concentrations of compounds and IBU in the cultures were 10, 50 and 100 μg/mL in all biological assays. The maximum concentration of DMSO in the individual assay was lower than 0.5%. These concentrations allowed us to avoid cell culture toxicity. The ibuprofen was selected as the reafference medicine generally used as nonsteroidal anti-inflammatory drugs (positive control). Additionally, control samples contained DMSO in the highest used dose (negative control) were prepared.

#### 4.2.2. Toxicity of Compounds **9**–**22**

Obtained PBMC were subjected to a 24 h culture (37 °C at 5% CO_2_ atmosphere) with the studied compounds. After stimulation, the cells were washed once (centrifuged at 400× *g* at 4 °C for 5 min) with PBS (phosphate-buffered saline; Biomed Lublin, Lublin, Poland) and then used for staining with fluorescein isothiocyanate (FITC)-labeled Annexin V and propidium iodide (Annexin V Apoptosis Detection Kit I, Becton Dickinson, Franklin Lakes, NJ, USA). This procedure has been described previously [43].

#### 4.2.3. Antiproliferative Activity of **12** and **19**–**22**

Lymphocyte proliferation was evaluated by BD Horizon™ Violet Proliferation Dye 450 (VPD450, BD Pharmingen, San Diego, CA, USA). This dye is used for flow cytometric monitoring of cell division. VPD450-stained cells were cultured for 72 h with the mitogen, phytohemagglutinin (PHA, Sigma-Aldrich, 1 µg/mL, positive control), and/or increasing concentrations of compounds **12** and **19**–**22** in DMSO (10, 50 and 100 μg/mL). Control samples contained DMSO in the highest used dose (negative control). Thereafter, culture tubes were centrifuged at 400× *g* at room temperature for 5 min and washed once in PBS. The cell palette was suspended in PBS and analyzed by flow cytometry (FACSCanto II flow cytometer, Becton Dickinson, Franklin Lakes, NJ, USA). Twenty thousand events were collected and analyzed with FlowJo software (v 7.6.1, Tree Star, Ashland, OR, USA).

#### 4.2.4. The Influence of **12** and **19**–**22** on Cytokine Production

The in vitro effect on cytokine production was measured by the enzyme-linked immunosorbent assay (ELISA). The assay was conducted as described earlier [41]. PBMC were cultured with lipopolysaccharide (LPS, from *E. coli*, O55:B5, Sigma-Aldrich, 1 μg/mL, positive control) and/or increasing concentrations of compounds. Cytokine levels (TNF-α, IL-6, IL-10 and IFN-γ) were measured by means of commercially available ELISA kits (DuoSet, BD Bioscience, San Diego, CA, USA) according to the manufacturer’s instructions. The samples were analyzed with iEMS Reader MF (Labsystems, Vanta, Finland). The contents of the analyzed cytokines were calculated by Ascent Software AscSW26 (London, UK).

#### 4.2.5. Data Analysis

Data was analyzed in Statistica 13.3 software (StatSoft, Cracow, Poland) and graphed in Excel 2016. All *p*-values represent nonparametric Mann−Whitney U test.

#### 4.2.6. Anthelmintic Activity of **10**–**22**

Due to their very low solubility in water, test compounds **10**–**22** were suspended in 60% glycerol. To obtain a homogeneous emulsion, sonication was performed using an ultrasonic homogenizer (Hielscher Ultrasound Technology). The culturing of nematodes and the methodology of testing nematocidal properties was developed by the Department of Biology and Genetics of the Medical University of Lublin (patent No. PL232918, Bogucka-Kocka A., Kołodziej P., 2019). The culturing was carried out for 4–5 days at room temperature. All development stages of the nematodes were observed.

The nematodes were then eluted with 0.6% NaCl into new, sterile 24-well plates. The tested derivatives were added to the nematode cultures in experimentally selected 5 concentrations: 0.2; 1.1; 3.3; 5.5; 11.1 µg/µL.

The anthelmintic activity was carried out according to the protocol described previously [24,38,48,50]. ANOVA and the Tukey test (GraphPad Prism 5.02 software, San Diego, CA, USA) was used for statistical analysis. Differences with *p* < 0.05 *, *p* < 0.01 ** and *p* < 0.001 *** were considered statistically significant [48,51].

## 5. Conclusions

Some biological properties of the 1,2,4-triazole derivatives **9**–**22** were experimentally evaluated. Most compounds (**11**–**22**) proved to be non-toxic to human PBMC. In anti-inflammatory studies, derivatives **19**–**22** showed higher activity than derivative **12** and previously tested compounds **11, 14**–**16**. The most active compound, **21,** acts in a number of ways: inhibiting the proliferation of PBMC and reducing the production of TNF-α and INF-γ in mitogen stimulated PBMC cultures. In general, the propenoic acid derivatives **17**–**22** had a stronger anti-inflammatory potential than compounds **11**–**16** differing in position of the double bond in the side chain.

Owing to their high anthelmintic potential, which was proved to be higher than that of the recommended drug albendazole, we have selected compounds **12** and **14** as potential candidates for further research. Derivative **12** also showed anti-inflammatory activity (by inhibiting TNF-α production), which constitutes an additional advantage. Interestingly, the clinically used anthelmintic drug ivermectin also has an anti-inflammatory effect [56]. Thanks to their anthelmintic and anti-inflammatory properties, the selected compounds may, in the future, be used in the treatment of parasitic diseases caused by intestinal nematodes in humans.

## 6. Patents

Part of the results of the work were used to prepare the Polish patent application No. P.434865.

## Data Availability

Data is available from the authors.

## Supplementary Materials

The following supporting information can be downloaded at: https://www.mdpi.com/article/10.3390/molecules27144488/s1, Figure S1: ^1^H NMR spectrum of compound **12** (in DMSO-d_6_). Figure S2: ^1^H NMR spectrum of compound **19** (in DMSO-d_6_). Figure S3: ^1^H NMR spectrum of compound **20** (in DMSO-d_6_). Figure S4: ^1^H NMR spectrum of compound **21** (in DMSO-d_6_). Figure S5: ^1^H NMR spectrum of compound **22** (in DMSO-d_6_). Figure S6. ^13^C NMR spectrum of compound **12** (in DMSO-d_6_). Figure S7. ^13^C NMR spectrum of compound **19** (in DMSO-d_6_). Figure S8. _13_C NMR spectrum of compound **20** (in DMSO-d_6_). Figure S9. ^13^C NMR spectrum of compound **21** (in DMSO-d_6_). Figure S10. ^13^C NMR spectrum of compound **22** (in DMSO-d_6_). Figure S11. HRMS data of compound **12**. Figure S12. HRMS data of compound **19**. Figure S13. HRMS data of compound **20**. Figure S14. HRMS data of compound **21**. Figure S15. HRMS data of compound **22**. Figure S16. HPLC data of compound **12**. Figure S17. HPLC data of compound **19**. Figure S18. HPLC data of compound **20**. Figure S19. HPLC data of compound **21**. Figure S20. HPLC data of compound **22**. Figure S21: Cell apoptosis of compounds **9**–**21** measured by flow cytometry.
