# Supplementary material for: Evaluation of Anthelmintic and Anti-Inflammatory Activity of 1,2,4-Triazole Derivatives
Source: Molecules. 2022 Jul 13;27(14):4488. doi: 10.3390/molecules27144488 (PMC9323247; doi:10.3390/molecules27144488)

Supplementary material

for publication

Evaluation of anthelmintic and anti-inflammatory activity of 1,2,4-triazole derivatives

Renata Paprocka ^[1,*], Przemysław Kołodziej ^[2,*], Małgorzata Wiese-Szadkowska ^[3], Anna Helmin-Basa [3] and Anna Bogucka-Kocka [2]

[1] Nicolaus Copernicus University in Toruń, Collegium Medicum in Bydgoszcz, Faculty of Pharmacy, Department of Organic Chemistry, Jurasza Str. 2, 85-089 Bydgoszcz, Poland; renata.bursa@cm.umk.pl

[2] Medical University in Lublin, Faculty of Pharmacy, Department of Biology and Genetics, Chodźki 4A, 20-093 Lublin, Poland; przemyslawkolodziej@umlub.pl (P.K), anna.bogucka-kocka@umlub.pl (A.B.-K.)

[3] Nicolaus Copernicus University in Toruń, Collegium Medicum in Bydgoszcz, Faculty of Pharmacy, Department of Immunology, M. Curie-Skłodowska Str. 9, 85-094 Bydgoszcz, Poland; mwiese@cm.umk.pl (M.W.-S.), a.helmin-basa@cm.umk.pl (A.H.-B.)

* Correspondence: renata.bursa@cm.umk.pl, przemyslawkolodziej@umlub.pl

^ These authors contributed equally to this work.

Characteristics of new compounds **12** and **19-22**

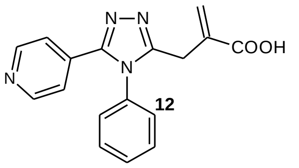

12

2-((4-(4-phenyl)-5-(pyridin-4-yl)-4*H*-1,2,4-triazol-3-yl)methyl)acrylic acid (**12**). Yield: 66%, m.p. 178-180°C. ^{1}H NMR (400 MHz, DMSO-d6): δ [ppm] = 3.53 (s, 2 H), 5.56 (s, 1 H); 6.11 (s, 1 H), 7.30 (m, 3 H), 7.45 (d, 3 H, *J* = 4.2 Hz), 7.87 (t, 1 H, *J* = 7.7 Hz), 7.95 (d, 1 H, *J* = 7.7 Hz), 8.27 (d, 1 H, *J* = 4.2 Hz), 12.57 (s, 1 H, COOH). ^{13}C NMR (75 MHz, DMSO-d6): δ [ppm] = 28.0, 124.2, 124.7, 127.6, 127.9, 129.6, 129.8, 135.5, 136.5, 137.6, 147.2, 149.4, 153.0, 154.4, 167.5. HR-MS m/z 307.1194 [M+ + 1] (calculated for $C_{17}H_{15}N_4O_2$: 307.1195). Elem. anal. for $C_{17}H_{14}N_4O_2$ calculated: C, 66.66; H, 4.61; N, 18.29%; found C, 66.37; H, 4.83; N, 17.91%.

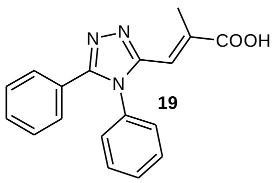

19

(*E*)-(4,5-diphenyl-4*H*-1,2,4-triazol-3-yl)-2-methylacrylic acid (**19**). Yield: 46.67%, m.p. 235-238°C. ^{1}H NMR (400 MHz, DMSO-d6): δ [ppm] = 2.43 (d, 3 H, *J* = 1.2 Hz), 6.85 (d, 1 H, *J* = 1.2 Hz), 7.33-7.44 (m, 5 H), 7.45-7.50 (m, 2 H), 7.56-7.62 (m, 3 H), 12.82 (sb, 1 H, COOH). ^{13}C NMR (100 MHz, DMSO-d6): δ [ppm] = 15.6, 119.5, 127.0, 128.6 (2x), 128.7 (2x), 129.0 (2x), 130.3, 130.6 (2x), 130.7, 134.3, 136.0, 152.3, 153.8, 168.7. HR-MS m/z 306.1240 [M+ + 1] (calculated for $C_{18}H_{16}N_3O_2$: 306.1243). Elem. anal. for $C_{18}H_{15}N_3O_2$ calculated: C, 67.66; H, 5.64; N, 12.46%; found C, 67.94; H, 5.72; N, 12.59%.

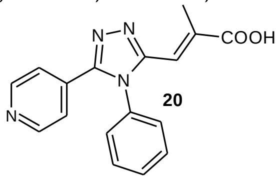

20

(*E*)-2-methyl-3-(4-phenyl-5-(pyridin-4-yl)-4*H*-1,2,4-triazol-3-yl)acrylic acid (**20**). Yield: 82.35%, m.p. 230-232ºC. ^{1}H NMR (400 MHz, DMSO-d6): δ [ppm] = 2.43 (d, 3 H, *J* = 1.6 Hz), 6.85 (d, 1 H, *J* = 1.6 Hz), 7.36-7.41 (m, 3 H), 7.50-7.56 (m, 3 H), 7.93 (t, 1 H, *J* = 7.6 Hz), 8.05 (d, 1 H, *J* = 7.6 Hz), 8.32 (d, 1 H, *J* = 4.2 Hz), 12.85 (sb, 1 H, COOH). ^{13}C NMR (100 MHz, DMSO-d6): δ [ppm] = 15.7, 119.4, 124.5, 125.0, 128.3 (2x), 129.9 (2x), 130.0, 135.0, 136.5, 137.7, 146.7, 149.5, 152.8, 152.9, 168.7. HR-MS m/z 307.1194 [M+ + 1] (calculated for $C_{17}H_{15}N_4O_2$: 307.1195). Elem. anal. for $C_{17}H_{14}N_4O_2$ calculated: C, 66.66; H, 4.61; N, 18.29%; found C, 66.30; H, 4.97; N, 17.91%.

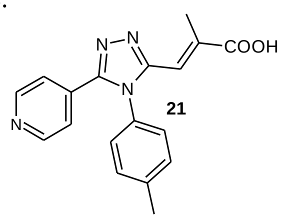

21

(*E*)-2-methyl-3-(5-(pyridin-4-yl)-4-*p*-tolyl-4*H*-1,2,4-triazol-3-yl)acrylic acid (**21**). Yield: 80.95%, m.p. 252-254ºC. ^{1}H NMR (400 MHz, DMSO-d6): δ [ppm] = 2.43 (s, 6 H), 6.86 (d, 1 H, *J* = 1.44 Hz), 7.34 (dd, 2 H, J_1 = 4.4 Hz, J_2 = 1.6Hz), 7.42 (d, 4 H, *J* = 3.2 Hz), 8.59 (dd, 2 H, J_1 = 4.4 Hz, J_2 = 1.6 Hz), 12.87 (sb, 1 H, COOH). ^{13}C NMR (100 MHz, DMSO-d6): δ [ppm] = 15.7, 21.3, 119.1, 122.3 (2x), 128.1 (2x), 131.2 (2x), 131.2, 134.4, 136.7 140.9, 150.6 (2x), 151.7, 153.2, 168.7. HR-MS m/z 321.1349 [M+ + 1] (calculated for $C_{18}H_{17}N_4O_2$: 321.1352). Elem. anal. for $C_{18}H_{16}N_4O_2$* 1/2 C_2H_5OH calculated: C, 66.52; H, 5.54; N, 16.39%; found C, 66.13; H, 5.23; N, 16.31%.

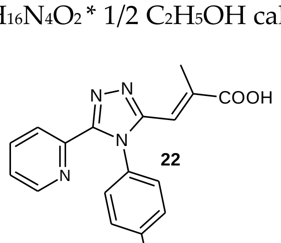

22

(*E*)-2-methyl-3-(5-(pyridin-2-yl)-4-*p*-tolyl-4*H*-1,2,4-triazol-3-yl)acrylic acid (**22**). Yield: 52.78%, m.p. 227-229°C. ^{1}H NMR (700 MHz, DMSO-d6): δ [ppm] = 2.38 (s, 3 H), 2.41 (d, 3 H, *J* = 1.68 Hz), 6.84 (d, 1 H, *J* = 1.68 Hz), 7.25 (d, 2 H, *J* = 7.7 Hz), 7.31 (d, 2 H, *J* = 7.7 Hz), 7.36-7.39 (m, 1 H), 7.91 (t, 1 H, *J* = 7.7 Hz), 8.00 (d, 1 H, *J* = 7.7 Hz), 8.35 (d, 1 H, *J* = 7.7 Hz), 12.79 (sb, 1 H, COOH). ^{13}C NMR (175 MHz, DMSO-d6): δ [ppm] = 15.7, 21.2, 119.5, 124.6, 124.9, 128.0 (2x), 130.3 (2x), 132.3, 136.4, 137.6, 139.5, 146.8, 149.6, 152.9, 153.0, 168.7. HR-MS m/z 321.1354 [M+ + 1] (calculated for $C_{18}H_{17}N_4O_2$: 321.1352). Elem. anal. for $C_{18}H_{16}N_4O_2$ calculated: C, 67.49; H, 5.03; N, 17.49%; found C, 67.37; H, 5.11; N, 17.43%.

Figure S1. ^{1}H NMR spectrum of compound **12** (in DMSO-d_6)

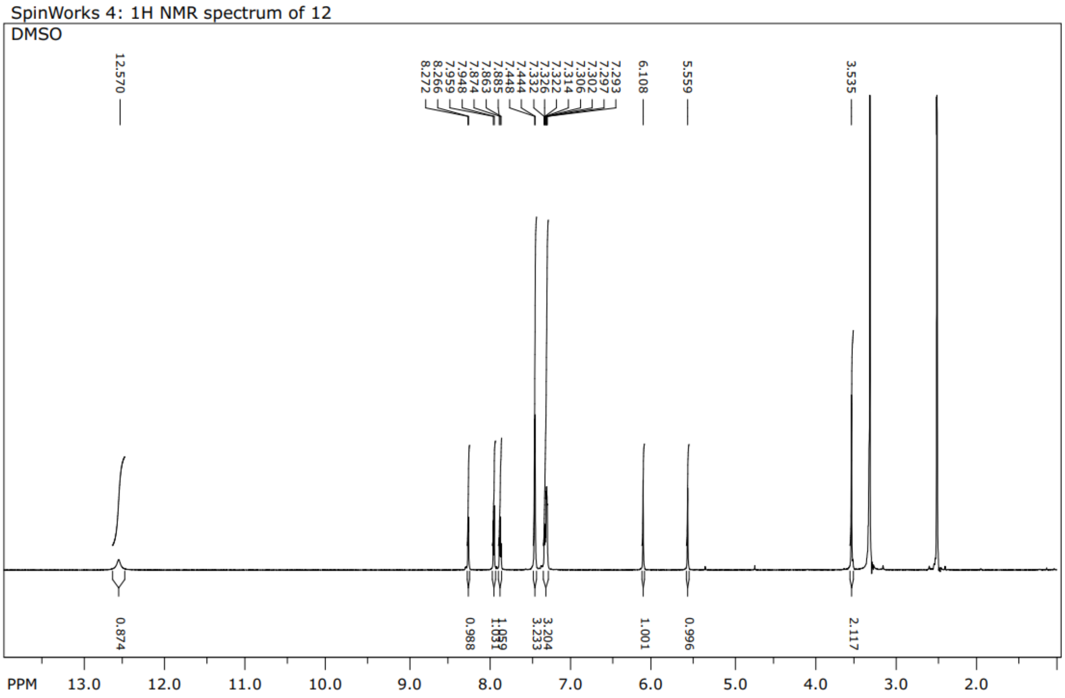

file: ...\moje badania\widma\1868 zw180\fid expt: <zg30>
transmitter freq.: 700.275602 MHz
time domain size: 169170 points
width: 14097.74 Hz = 20.1317 ppm = 0.083335 Hz/pt
number of scans: 8

freq. of 0 ppm: 700.270026 MHz
processed size: 16384 complex points
LB: 0.000 GF: 0.0000
Hz/cm: 364.391 ppm/cm: 0.52035

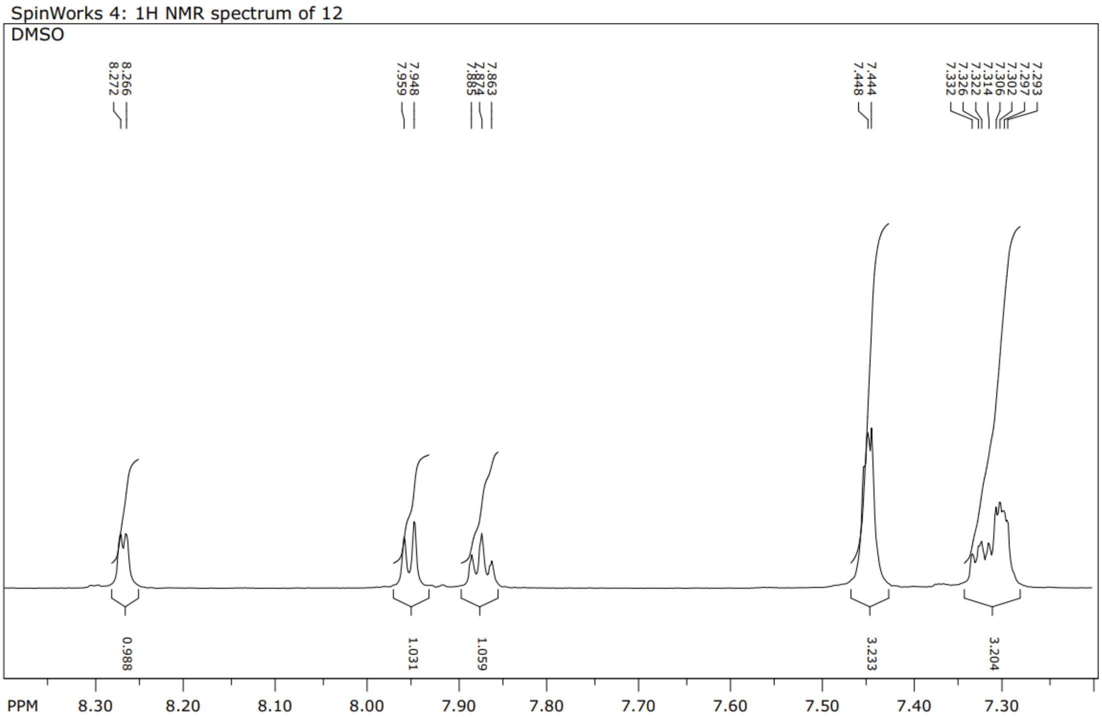

file: ...\moje badania\widma\1868 zw180\fid expt: <zg30>
transmitter freq.: 700.275602 MHz
time domain size: 169170 points
width: 14097.74 Hz = 20.1317 ppm = 0.083335 Hz/pt
number of scans: 8

freq. of 0 ppm: 700.270026 MHz
processed size: 16384 complex points
LB: 0.000 GF: 0.0000
Hz/cm: 33.705 ppm/cm: 0.04813

Figure S2. ^{1}H NMR spectrum of compound **19** (in DMSO-d_6)

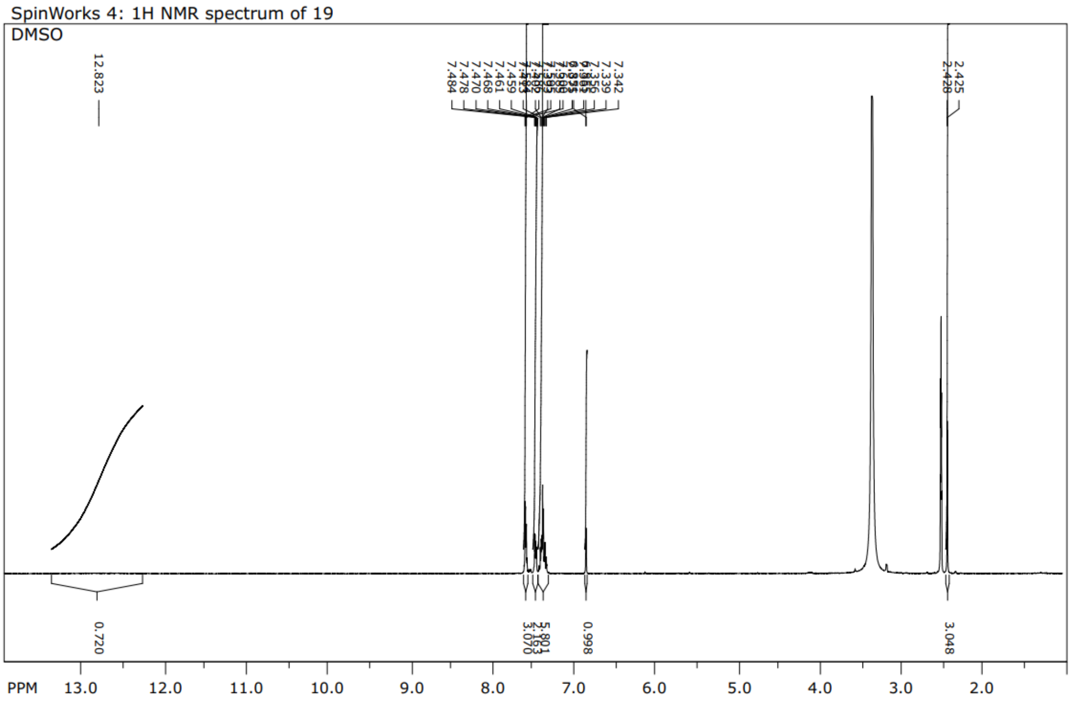

file: ...:\moje badania\widma\9004 361T\fid expt: <zg30>
transmitter freq.: 400.133401 MHz
time domain size: 64100 points
width: 8012.82 Hz = 20.0254 ppm = 0.125005 Hz/pt
number of scans: 16

freq. of 0 ppm: 400.130002 MHz
processed size: 65536 complex points
LB: 0.000 GF: 0.0000
Hz/cm: 207.564 ppm/cm: 0.51874

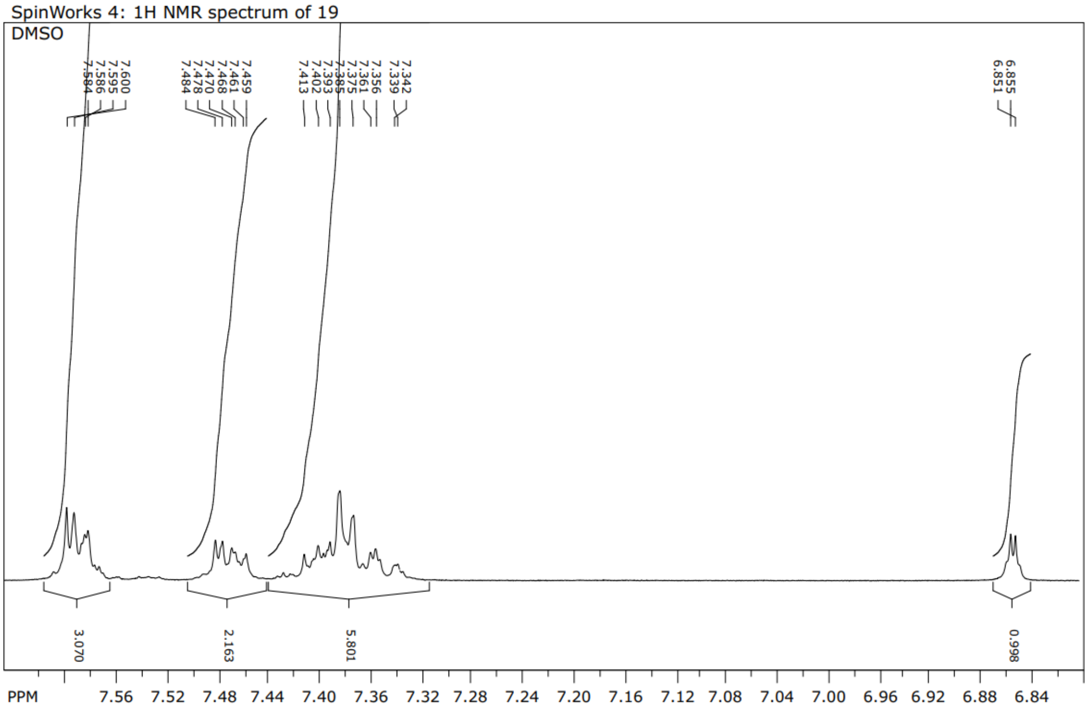

file: ...:\moje badania\widma\9004 361T\fid expt: <zg30>
transmitter freq.: 400.133401 MHz
time domain size: 64100 points
width: 8012.82 Hz = 20.0254 ppm = 0.125005 Hz/pt
number of scans: 16

freq. of 0 ppm: 400.130002 MHz
processed size: 65536 complex points
LB: 0.000 GF: 0.0000
Hz/cm: 13.603 ppm/cm: 0.03400

Figure S3. ^{1}H NMR spectrum of compound **20** (in DMSO-d_6)

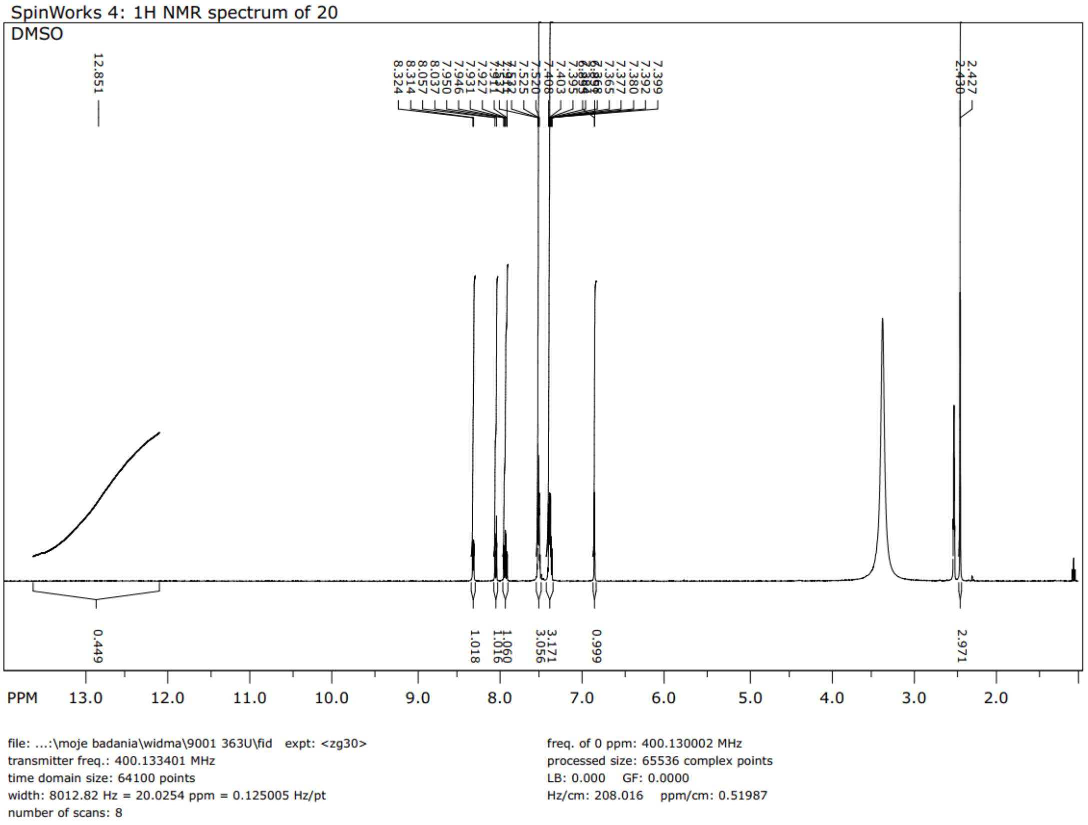

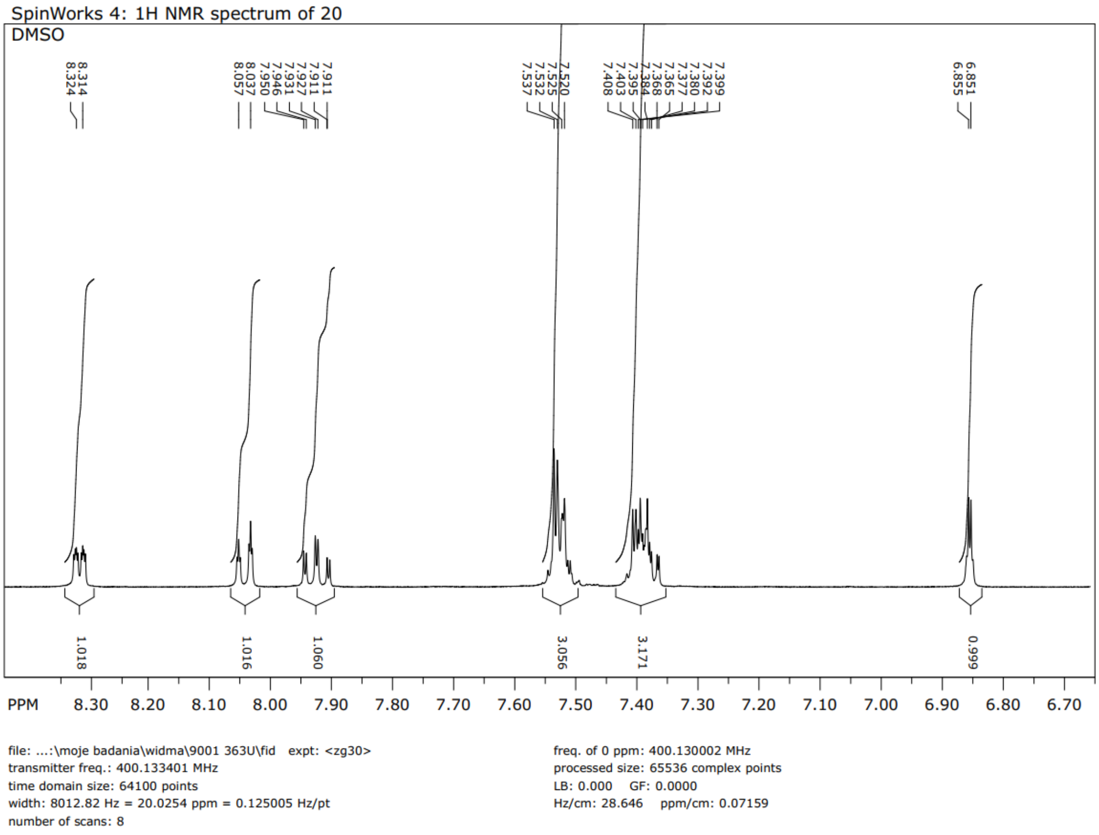

Figure S4. ^{1}H NMR spectrum of compound **21** (in DMSO-d_6)

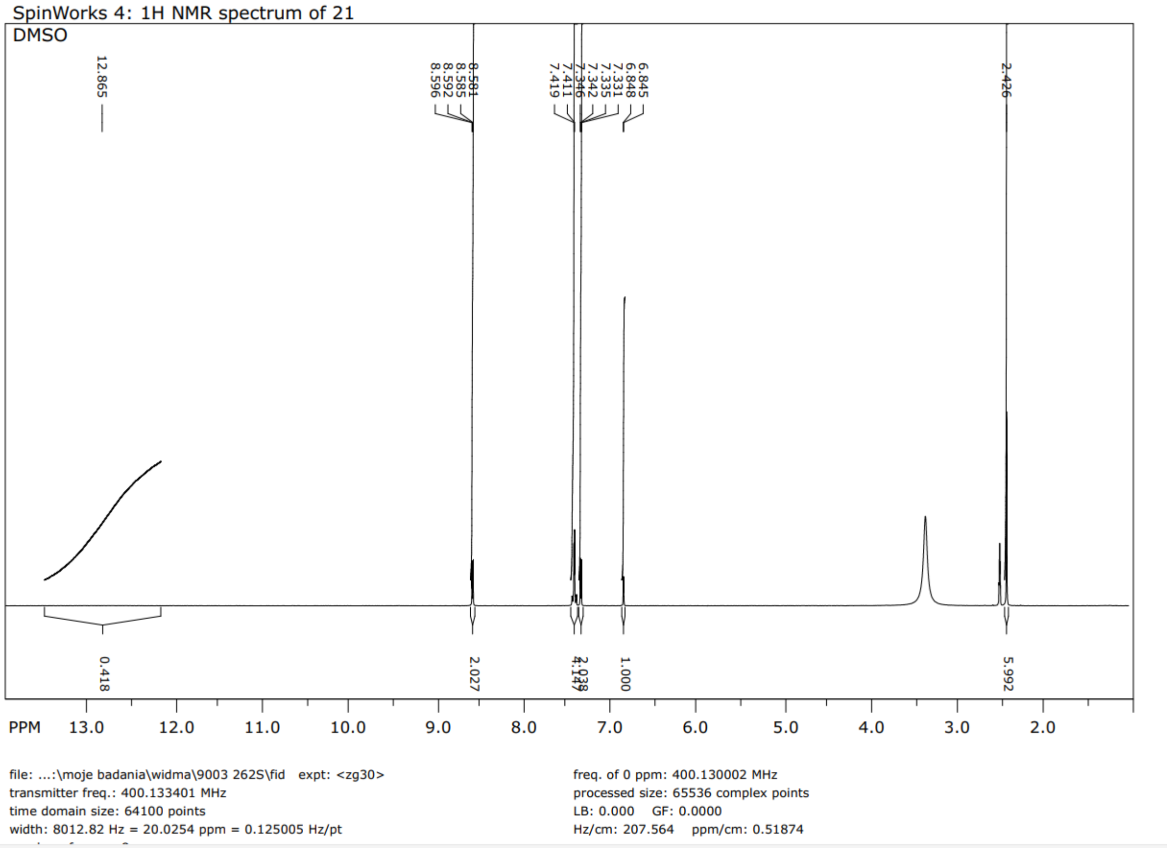

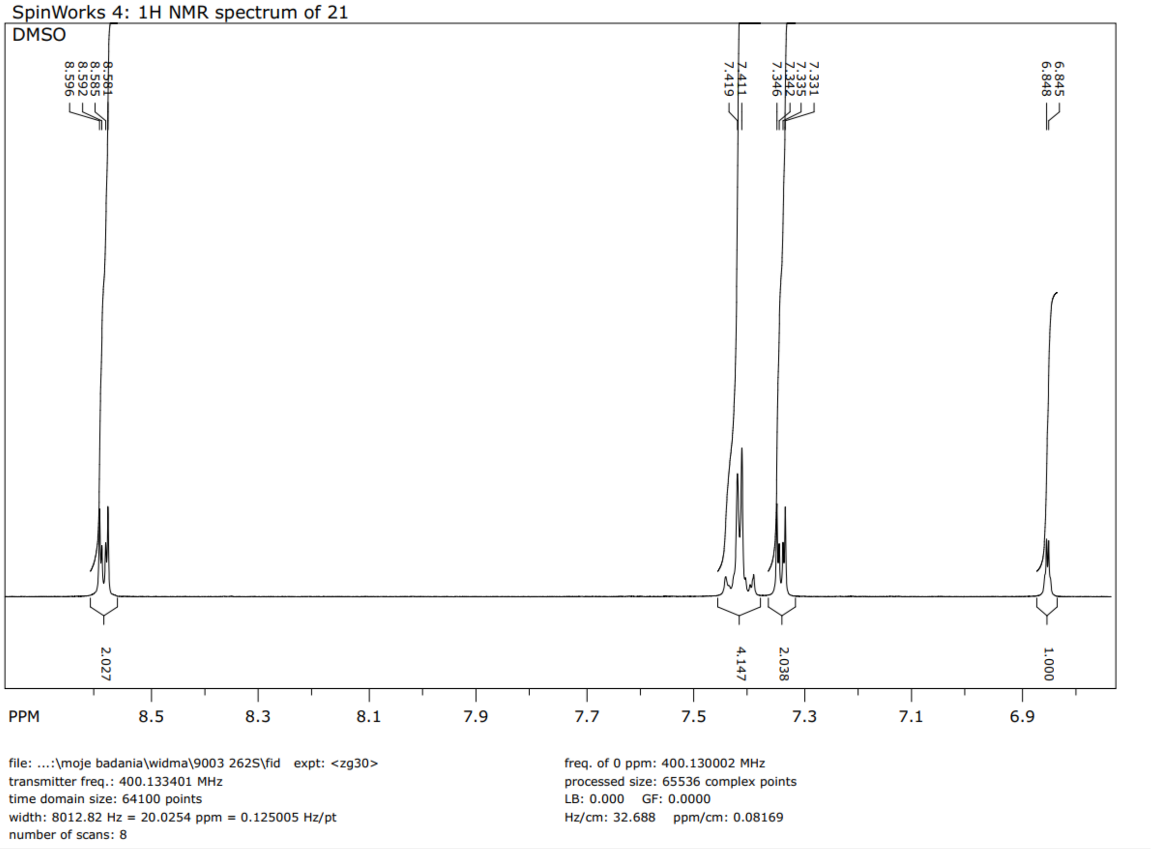

Figure S5. ^{1}H NMR of compound **22** (in DMSO-d_6)

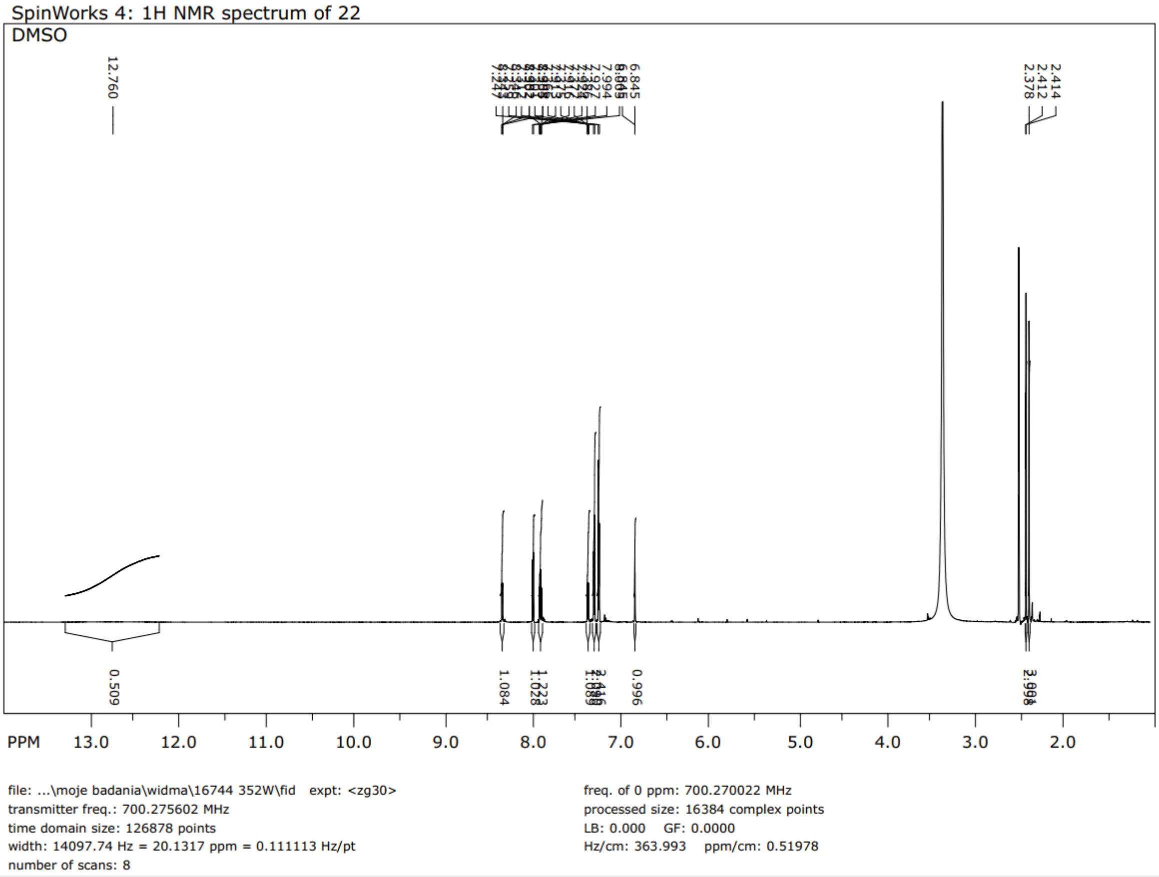

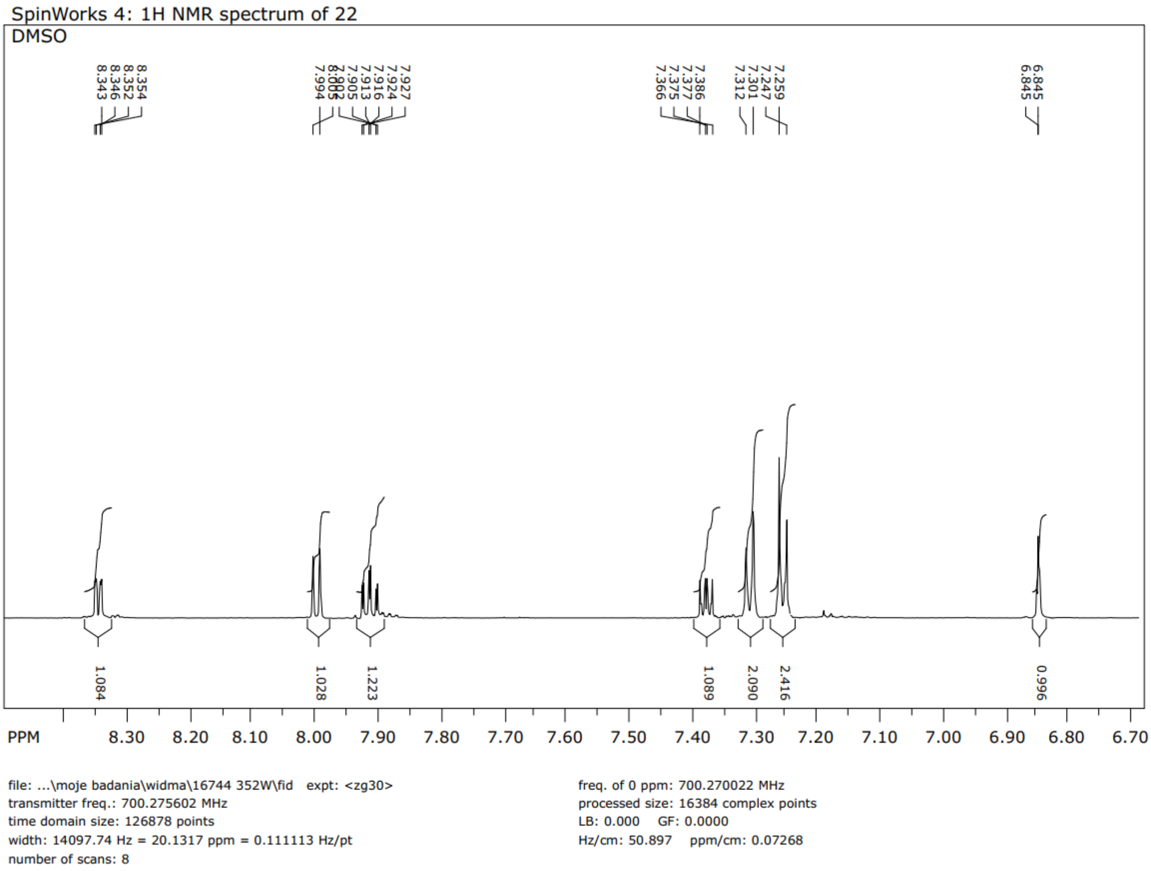

Figure S6. ^{13}C NMR spectrum of compound **12** (in DMSO-d$_6$)

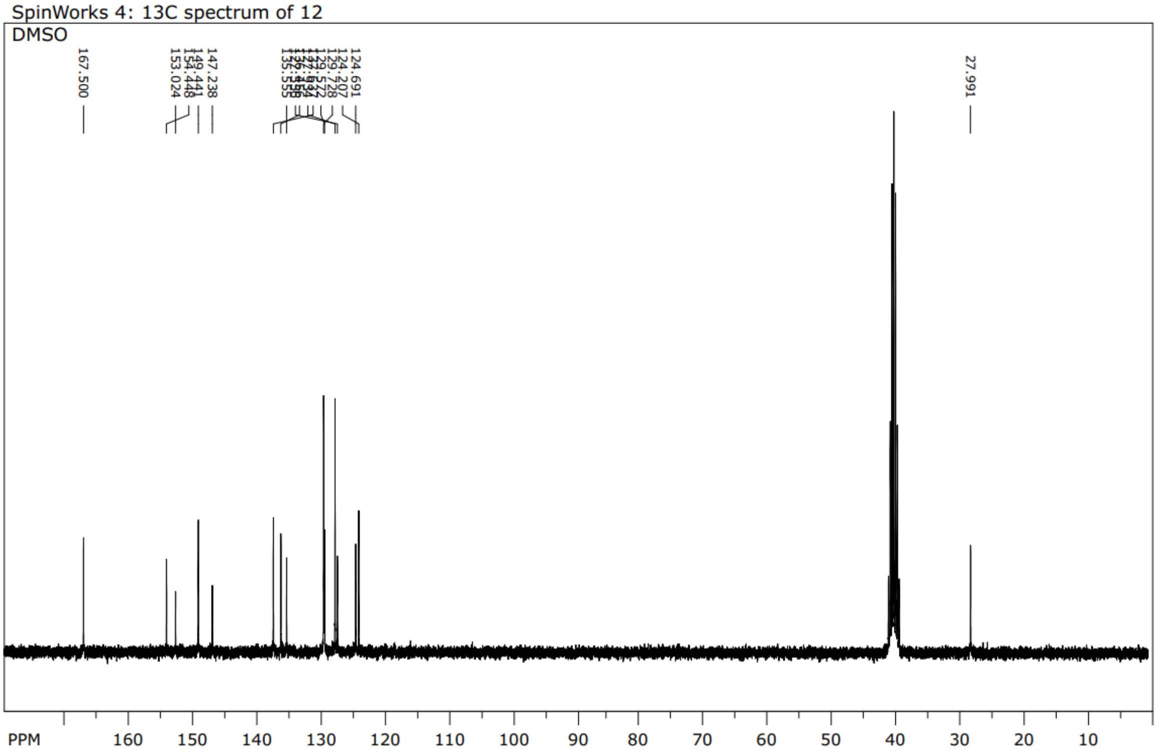

file: ...adania\DANEzUMCS\NMR HIKMOP\13\fid expt: <zgpg30>
transmitter freq.: 75.524303 MHz
time domain size: 65536 points
width: 18115.94 Hz = 239.8690 ppm = 0.276427 Hz/pt
number of scans: 256

freq. of 0 ppm: 75.516776 MHz
processed size: 65536 complex points
LB: 0.500 GF: 0.0000
Hz/cm: 543.478 ppm/cm: 7.19607

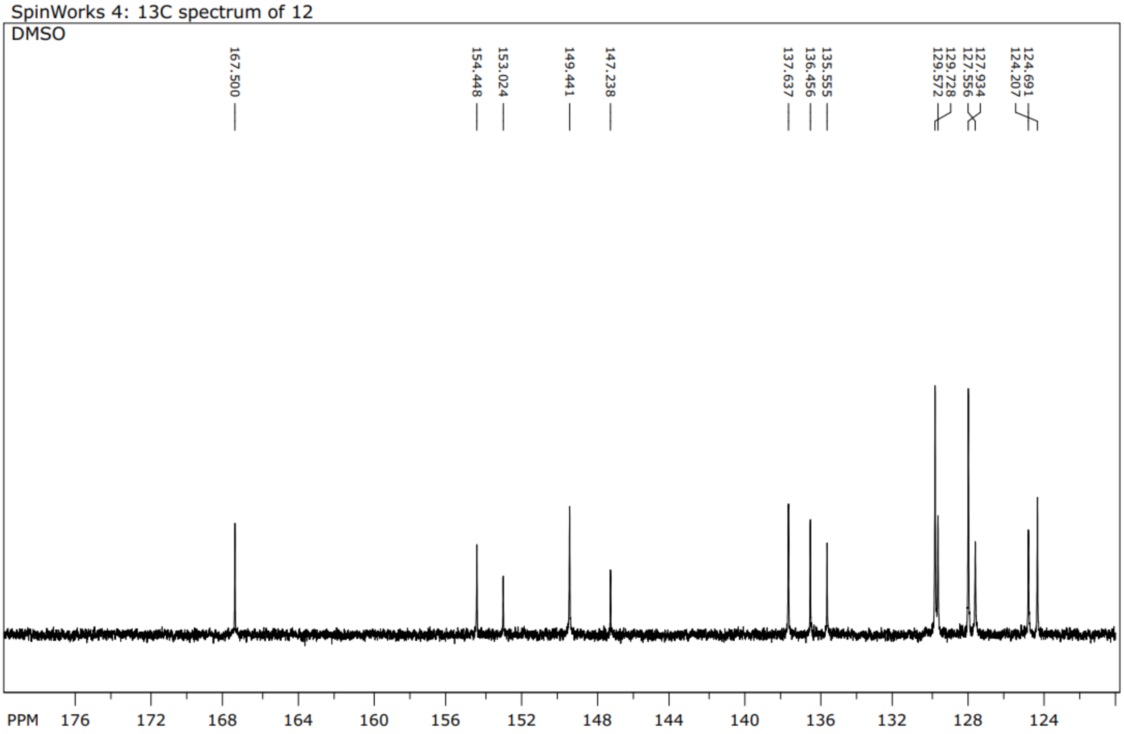

file: ...adania\DANEzUMCS\NMR HIKMOP\13\fid expt: <zgpg30>
transmitter freq.: 75.524303 MHz
time domain size: 65536 points
width: 18115.94 Hz = 239.8690 ppm = 0.276427 Hz/pt
number of scans: 256

freq. of 0 ppm: 75.516776 MHz
processed size: 65536 complex points
LB: 0.500 GF: 0.0000
Hz/cm: 181.159 ppm/cm: 2.39869

Figure S7. ^{13}C NMR spectrum of compound **19** (in DMSO-d_6)

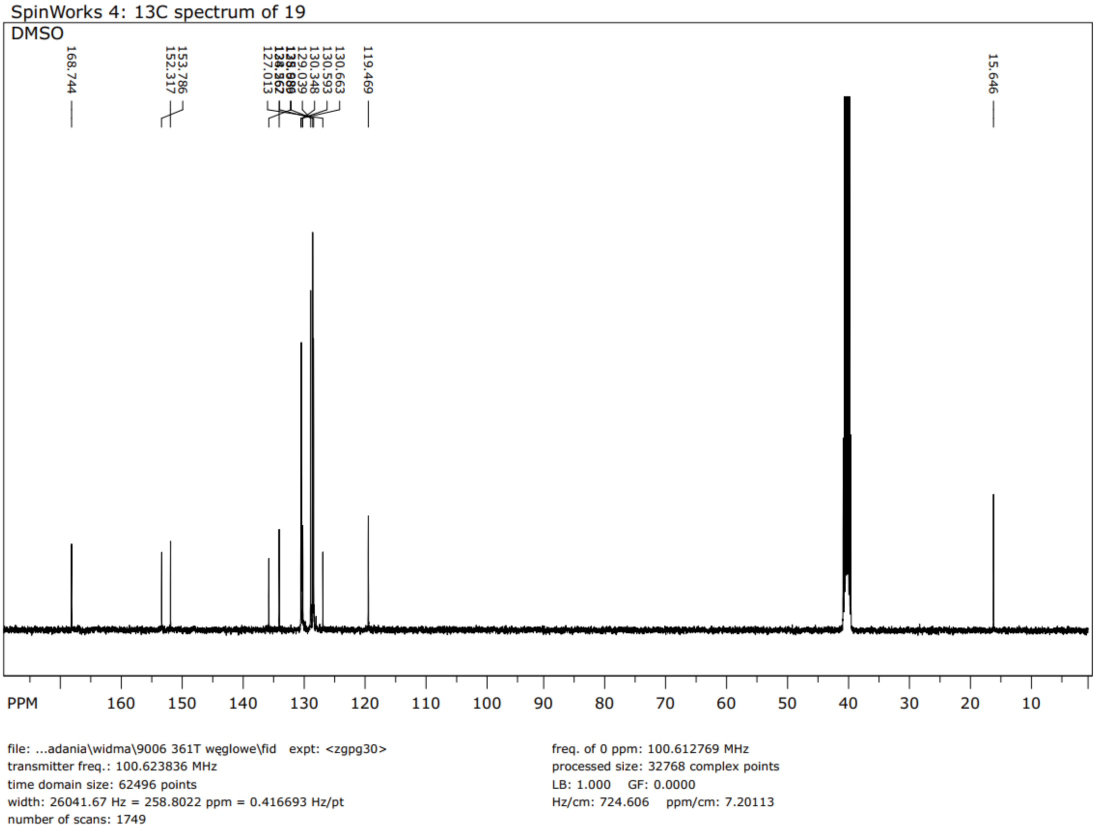

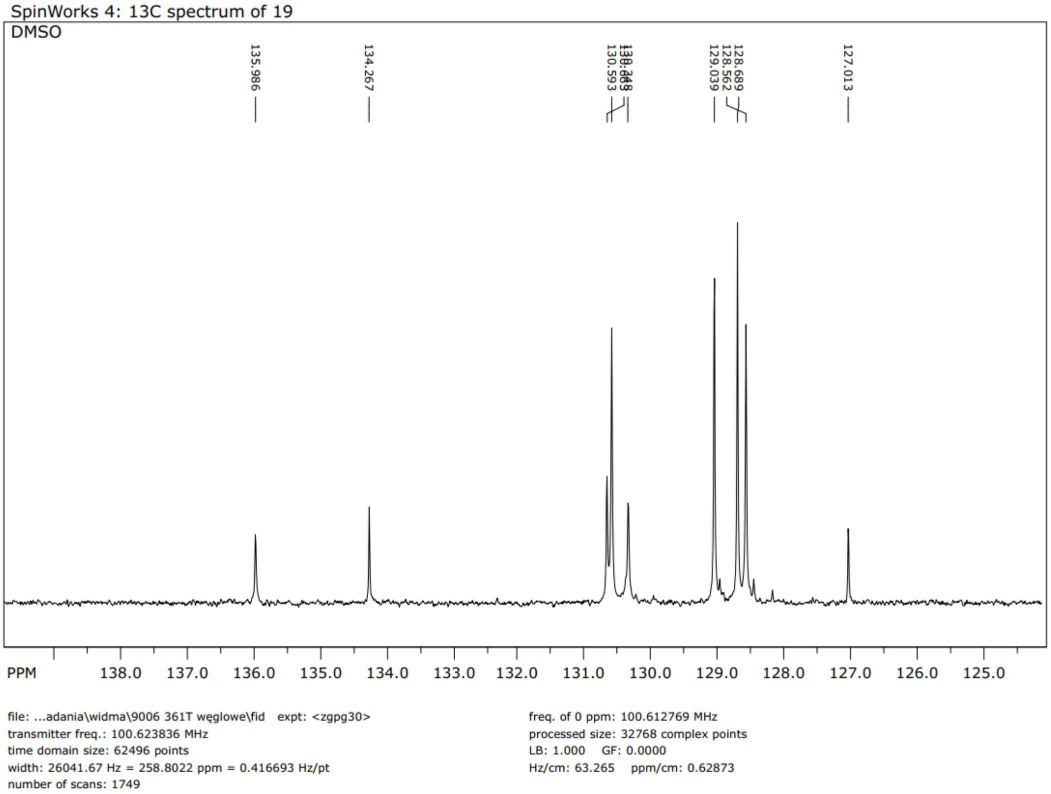

Figure S8. ^{13}C NMR spectrum of compound **20** (in DMSO-d$_6$)

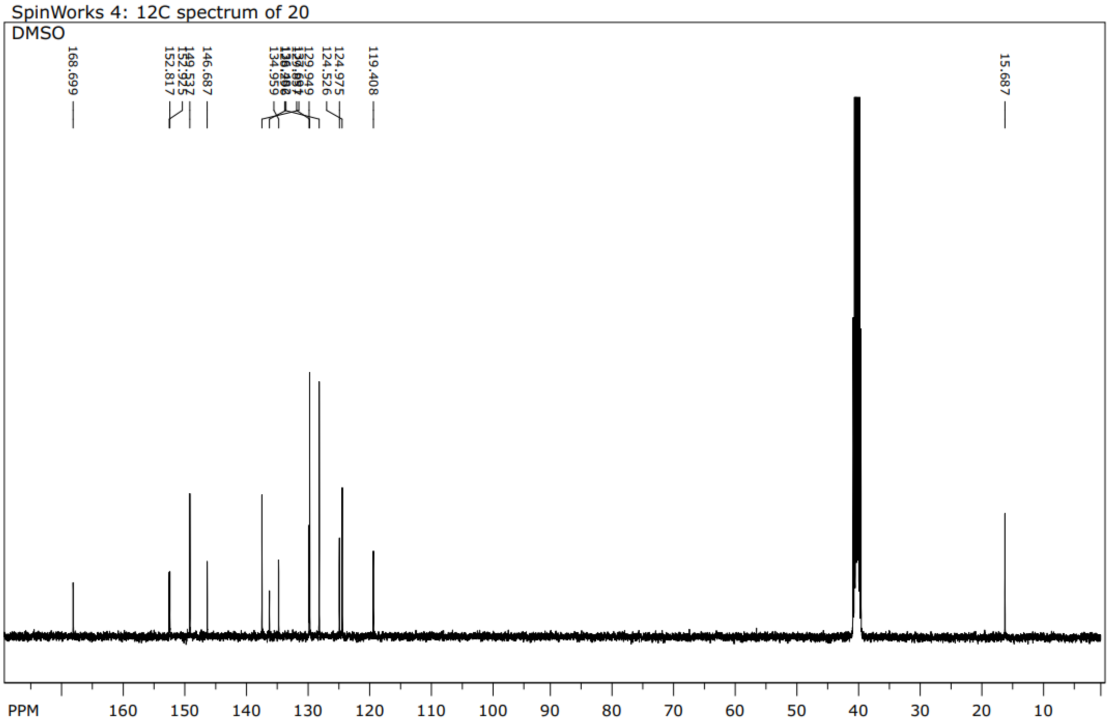

file: ...adania\widma\9002 363U węglowe\fid expt: <zgpg30>
transmitter freq.: 100.623836 MHz
time domain size: 62496 points
width: 26041.67 Hz = 258.8022 ppm = 0.416693 Hz/pt
number of scans: 2701

freq. of 0 ppm: 100.612769 MHz
processed size: 32768 complex points
LB: 1.000 GF: 0.0000
Hz/cm: 724.606 ppm/cm: 7.20113

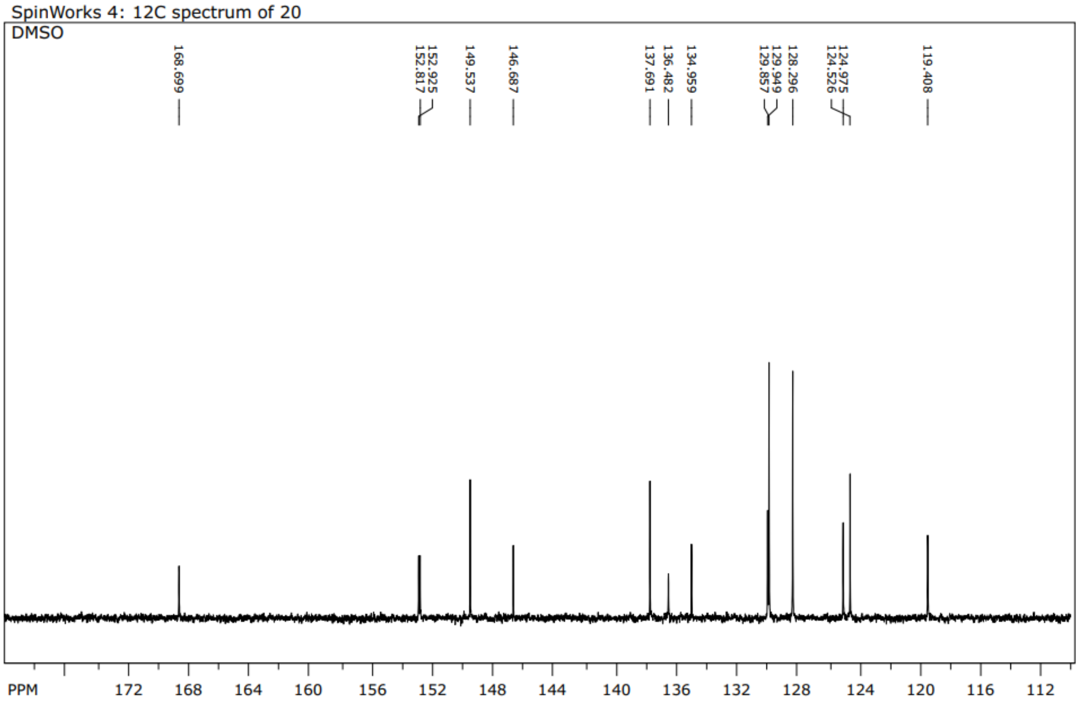

file: ...adania\widma\9002 363U węglowe\fid expt: <zgpg30>
transmitter freq.: 100.623836 MHz
time domain size: 62496 points
width: 26041.67 Hz = 258.8022 ppm = 0.416693 Hz/pt
number of scans: 2701

freq. of 0 ppm: 100.612769 MHz
processed size: 32768 complex points
LB: 1.000 GF: 0.0000
Hz/cm: 282.486 ppm/cm: 2.80735

Figure S9. ^{13}C NMR spectrum of compound **21** (in DMSO-d_6)

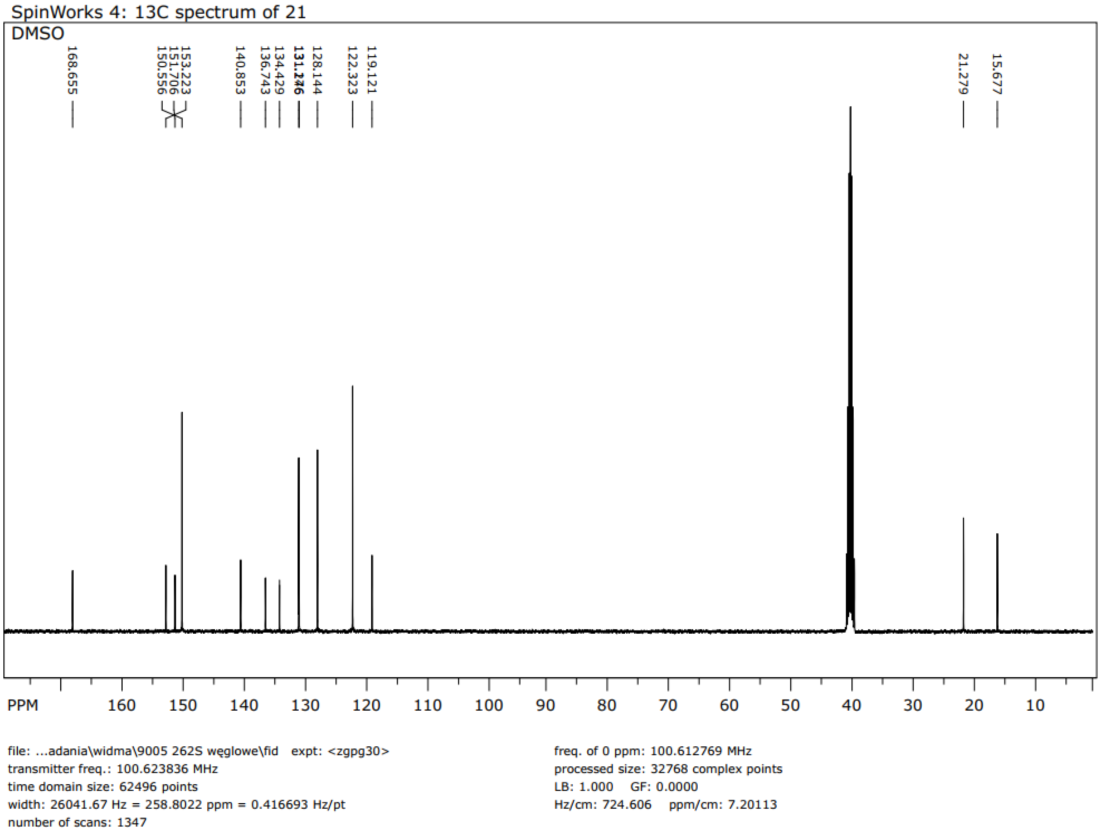

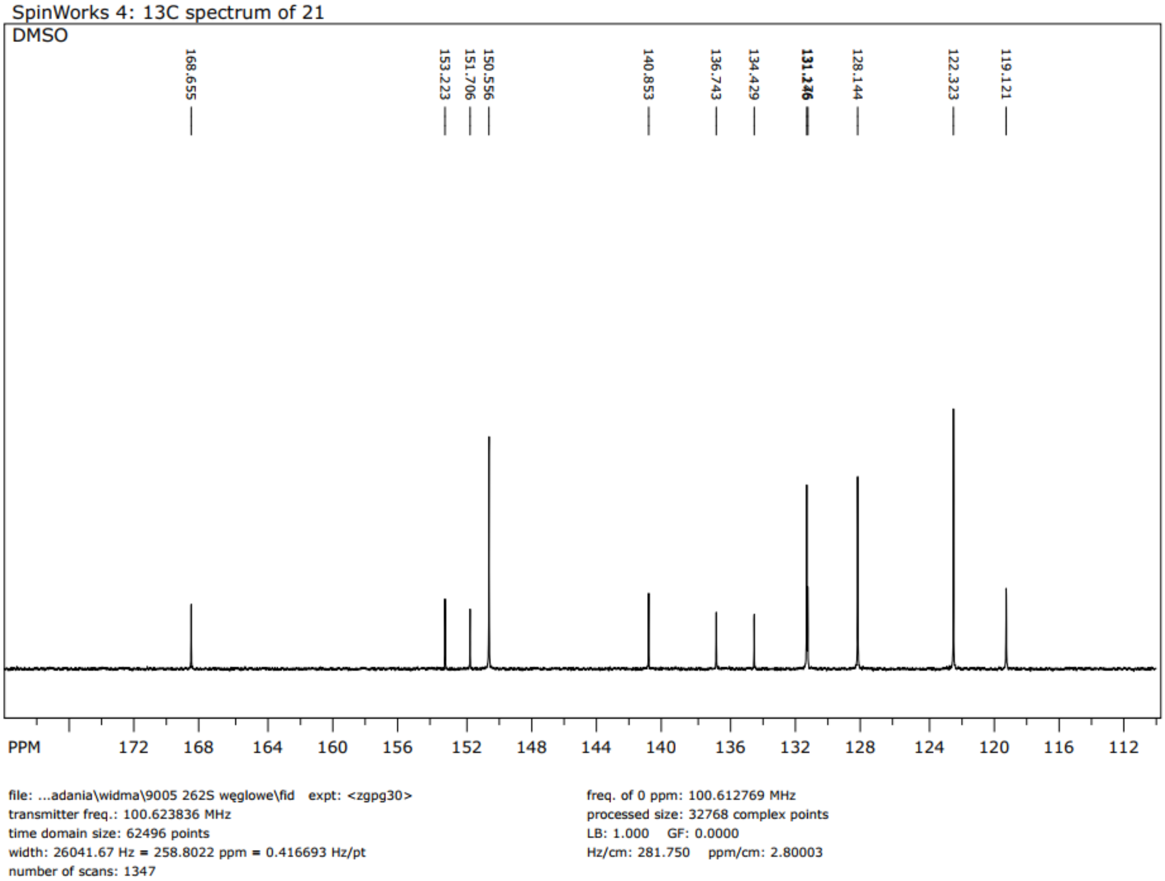

Figure S10. ^{13}C NMR spectrum of compound **22** (in DMSO-d_6)

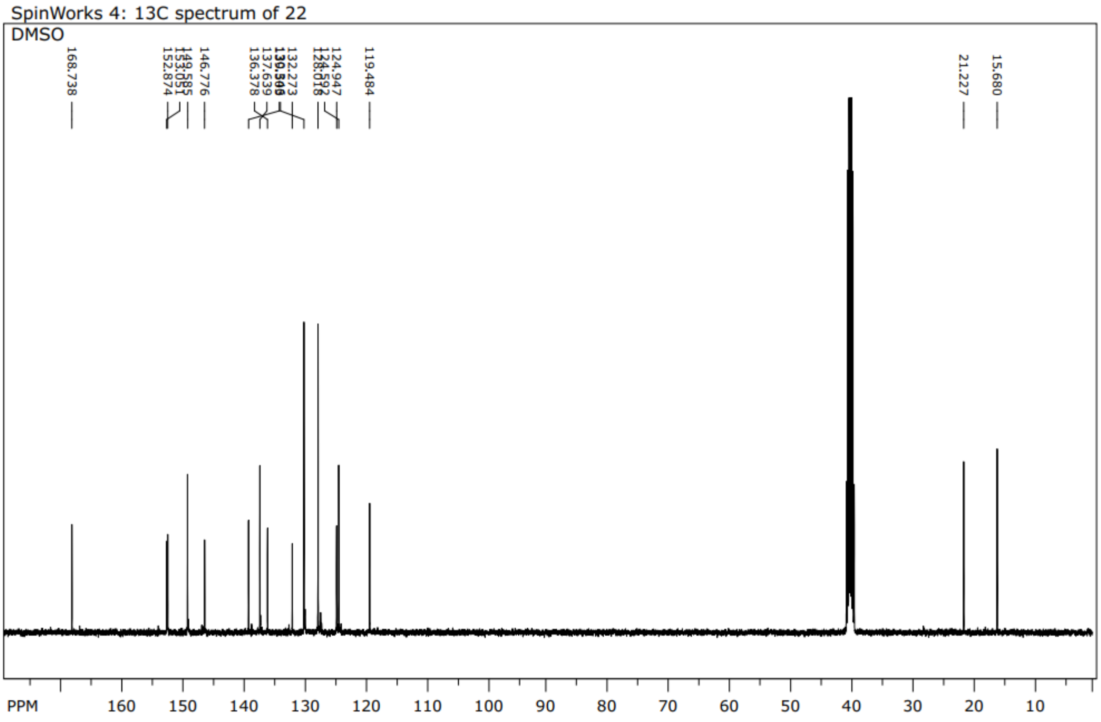

file: ...adania\widma\8997 252W węglowe\fid expt: <zgpg30>
transmitter freq.: 100.623836 MHz
time domain size: 62496 points
width: 26041.67 Hz = 258.8022 ppm = 0.416693 Hz/pt
number of scans: 1303

freq. of 0 ppm: 100.612769 MHz
processed size: 32768 complex points
LB: 1.000 GF: 0.0000
Hz/cm: 724.606 ppm/cm: 7.20113

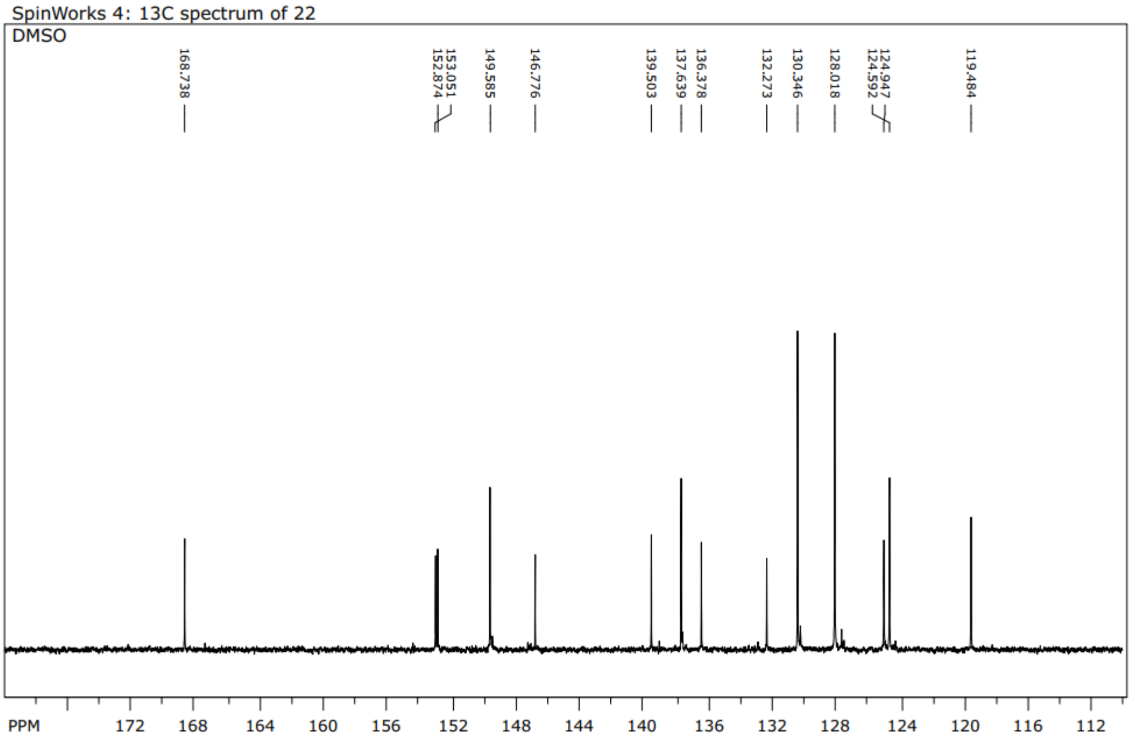

file: ...adania\widma\8997 252W węglowe\fid expt: <zgpg30>
transmitter freq.: 100.623836 MHz
time domain size: 62496 points
width: 26041.67 Hz = 258.8022 ppm = 0.416693 Hz/pt
number of scans: 1303

freq. of 0 ppm: 100.612769 MHz
processed size: 32768 complex points
LB: 1.000 GF: 0.0000
Hz/cm: 281.750 ppm/cm: 2.80003

Figure S11. HRMS data of compound **12**

Elemental Composition Report **Page 1**

Single Mass Analysis
Tolerance = 5.0 PPM / DBE: min = -1.5, max = 90.0
Element prediction: Off
Number of isotope peaks used for i-FIT = 9

Monoisotopic Mass, Even Electron Ions
175 formula(e) evaluated with 2 results within limits (all results (up to 1000) for each mass)
Elements Used:
C: 0-35 H: 0-30 N: 0-5 O: 0-10

220704_probka_12_A 22 (0.240) Cm (22:24-6:8) TOF MS ES+ 1.62e+006

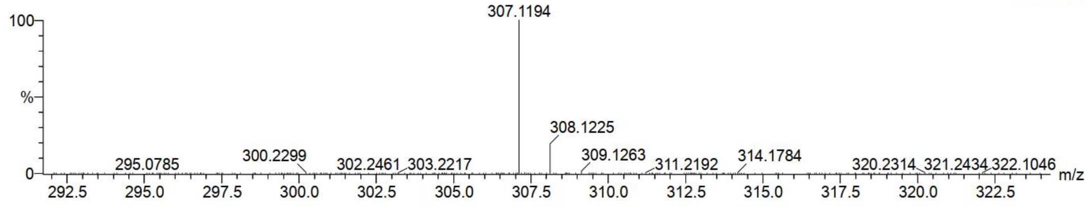

Minimum:			-1.5
Maximum:	5.0	5.0	90.0

Figure S12. HRMS data of compound **19**

Single Mass Analysis
Tolerance = 5.0 PPM / DBE: min = -1.5, max = 90.0
Element prediction: Off
Number of isotope peaks used for i-FIT = 9

Monoisotopic Mass, Even Electron Ions
174 formula(e) evaluated with 1 results within limits (all results (up to 1000) for each mass)
Elements Used:
C: 0-35 H: 0-30 N: 0-5 O: 0-10

220704_probka_19TA 29 (0.311) Cm (29:33-3:7) TOF MS ES+ 4.07e+006

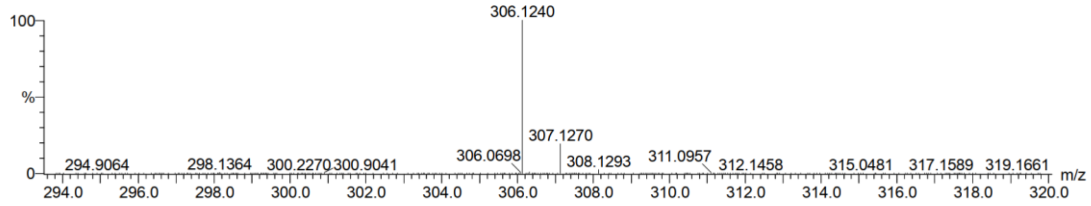

Minimum:			-1.5
Maximum:	5.0	5.0	90.0

Mass	Calc. Mass	mDa	PPM	DBE	i-FIT	Norm	Conf(%)	Formula

Figure S13. HRMS data of compound **20**

Elemental Composition Report **Page 1**

Single Mass Analysis
Tolerance = 5.0 PPM / DBE: min = -1.5, max = 90.0
Element prediction: Off
Number of isotope peaks used for i-FIT = 9

Monoisotopic Mass, Even Electron Ions
175 formula(e) evaluated with 2 results within limits (all results (up to 1000) for each mass)
Elements Used:
C: 0-35 H: 0-30 N: 0-5 O: 0-10
220704_probka_20UA 26 (0.285) Cm (26:31-3:6)

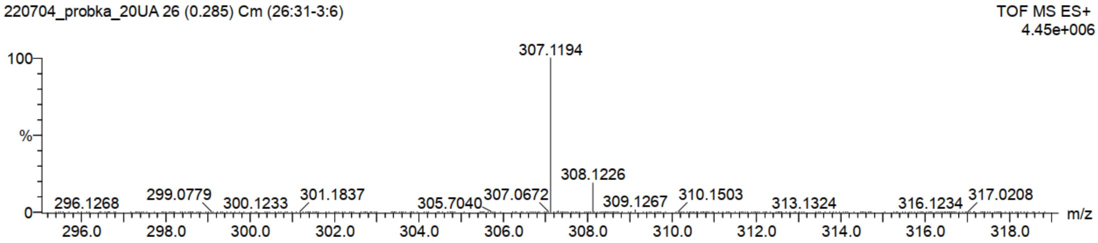

Minimum:				-1.5				
Maximum:		5.0	5.0	90.0				
Mass	Calc. Mass	mDa	PPM	DBE	i-FIT	Norm	Conf(%)	Formula
307.1194	307.1195	-0.1	-0.3	12.5	1246.6	0.024	97.67	C17 H15 N4 O2
	307.1182	1.2	3.9	7.5	1250.3	3.759	2.33	C16 H19 O6

Figure S14. HRMS data of compound **21**

Elemental Composition Report **Page 1**

Single Mass Analysis
Tolerance = 5.0 PPM / DBE: min = -1.5, max = 90.0
Element prediction: Off
Number of isotope peaks used for i-FIT = 9

Monoisotopic Mass, Even Electron Ions
176 formula(e) evaluated with 2 results within limits (all results (up to 1000) for each mass)
Elements Used:
C: 0-35 H: 0-30 N: 0-5 O: 0-10
220704_probka_21SA 24 (0.257) Cm (24:26-2:4)

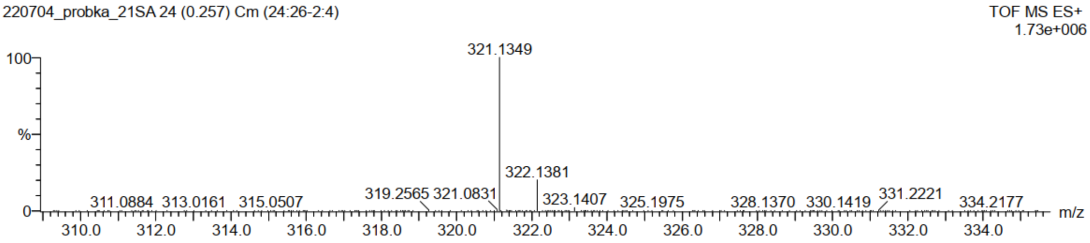

Minimum:				-1.5				
Maximum:		5.0	5.0	90.0				
Mass	Calc. Mass	mDa	PPM	DBE	i-FIT	Norm	Conf(%)	Formula
321.1349	321.1352	-0.3	-0.9	12.5	796.7	0.019	98.12	C18 H17 N4 O2
	321.1338	1.1	3.4	7.5	800.7	3.976	1.88	C17 H21 O6

Figure S15. HRMS data of compound **22**

Elemental Composition Report

Single Mass Analysis

Tolerance = 5.0 PPM / DBE: min = -1.5, max = 90.0
Element prediction: Off
Number of isotope peaks used for i-FIT = 9

Monoisotopic Mass, Even Electron Ions
176 formula(e) evaluated with 2 results within limits (all results (up to 1000) for each mass)
Elements Used:
C: 0-35 H: 0-30 N: 0-5 O: 0-10

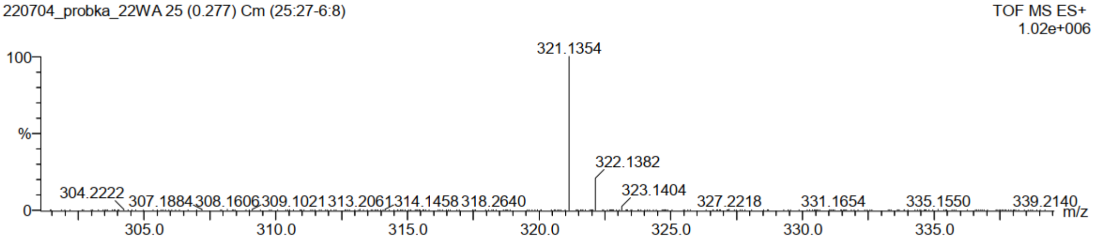

Minimum:				-1.5
Maximum:		5.0	5.0	90.0

Mass	Calc. Mass	mDa	PPM	DBE	i-FIT	Norm	Conf(%)	Formula
321.1354	321.1352	0.2	0.6	12.5	635.2	0.015	98.55	C18 H17 N4 O2
	321.1338	1.6	5.0	7.5	639.5	4.232	1.45	C17 H21 O6

Figure S16. HPLC data of **12***

SHIMADZU LabSolutions

Analysis Report

<Sample Information>

Sample Name	: 12M		
Sample ID	:		
Data Filename	: 12M.lcd		
Method Filename	: metoda.lcm		
Batch Filename	: batch.lcb		
Vial #	: 0-6	Sample Type	: Unknown
Injection Volume	: 10 uL		
Date Acquired	: 2022-07-08 10:11:18	Acquired by	: System Administrator
Date Processed	: 2022-07-08 12:20:47	Processed by	: System Administrator

<Chromatogram>

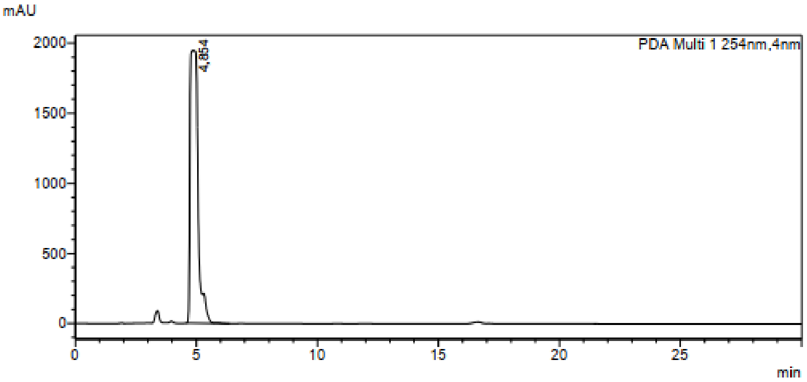

Peak Table

PDA Ch1 254nm

Peak#	Ret. Time	Area	Height	Conc.	Unit	Mark	Name
1	4.854	43943817	1943964	0.000		M	
Total		43943817	1943964				

*Chromatograph Shimadzu (pump: LC30AD Nexera X2, autosampler: SIL30AC Nexera X2, PDA detector: SPDM20A, thermostat: CTO20AC), mobile phase: MeOH/ H_2O/FA (formic acid) 50/50/0.1 (v/v/v). stationary phase: Kinetex C18 2.6 µm 150 x 4.6 mm, mobile phase flow: 0.8 mL / min, temp: 20°C, detection: PDA (channel 1: 254 nm + full spectrum in the range 190-800 nm).

Figure S17. HPLC data of **19***

SHIMADZU LabSolutions **Analysis Report**

<Sample Information>

Sample Name	: 19T		
Sample ID	:		
Data Filename	: 19T.lcd		
Method Filename	: metoda.lcm		
Batch Filename	: batch.lcb		
Vial #	: 0-2	Sample Type	: Unknown
Injection Volume	: 10 uL		
Date Acquired	: 2022-07-06 14:32:30	Acquired by	: System Administrator
Date Processed	: 2022-07-06 17:10:58	Processed by	: System Administrator

<Chromatogram>

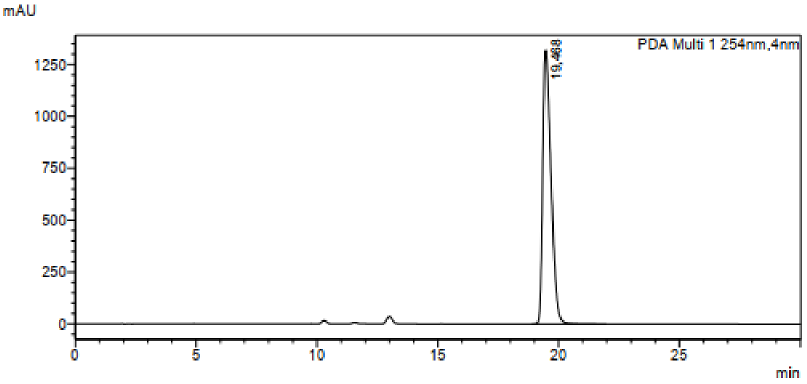

Peak Table

PDA Ch1 254nm

Peak#	Ret. Time	Area	Height	Conc.	Unit	Mark	Name
1	19.468	31530873	1317100	0.000			
Total		31530873	1317100				

*Chromatograph Shimadzu (pump: LC30AD Nexera X2, autosampler: SIL30AC Nexera X2, PDA detector: SPDM20A, thermostat: CTO20AC), mobile phase: MeOH/ H_2O/FA (formic acid) 50/50/0.1 (v/v/v). stationary phase: Kinetex C18 2.6 µm 150 x 4.6 mm, mobile phase flow: 0.8 mL / min, temp: 20°C, detection: PDA (channel 1: 254 nm + full spectrum in the range 190-800 nm).

Figure S18. HPLC data of **20***

2022-07-06 17:17:29 Page 1 / 1

SHIMADZU LabSolutions **Analysis Report**

<Sample Information>

Sample Name	: 20U		
Sample ID	:		
Data Filename	: 20U.lcd		
Method Filename	: metoda.lcm		
Batch Filename	: batch.lcb		
Vial #	: 0-3	Sample Type	: Unknown
Injection Volume	: 10 uL		
Date Acquired	: 2022-07-06 15:03:09	Acquired by	: System Administrator
Date Processed	: 2022-07-06 17:15:16	Processed by	: System Administrator

<Chromatogram>

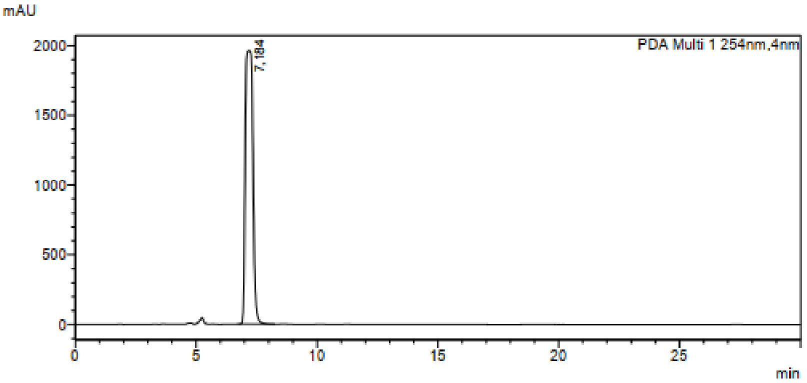

Peak Table

PDA Ch1 254nm

Peak#	Ret. Time	Area	Height	Conc.	Unit	Mark	Name
1	7.184	42926974	1959997	0.000		M	
Total		42926974	1959997				

*Chromatograph Shimadzu (pump: LC30AD Nexera X2, autosampler: SIL30AC Nexera X2, PDA detector: SPDM20A, thermostat: CTO20AC), mobile phase: MeOH/ H_2O/FA (formic acid) 50/50/0.1 (v/v/v). stationary phase: Kinetex C18 2.6 µm 150 x 4.6 mm, mobile phase flow: 0.8 mL / min, temp: 20°C, detection: PDA (channel 1: 254 nm + full spectrum in the range 190-800 nm).

Figure S19. HPLC data of **21***

SHIMADZU LabSolutions

Analysis Report

<Sample Information>

Sample Name	: 21S		
Sample ID	:		
Data Filename	: 21S.lcd		
Method Filename	: metoda.lcm		
Batch Filename	: batch.lcb		
Vial #	: 0-4	Sample Type	: Unknown
Injection Volume	: 10 uL		
Date Acquired	: 2022-07-06 15:33:48	Acquired by	: System Administrator
Date Processed	: 2022-07-06 17:10:42	Processed by	: System Administrator

<Chromatogram>

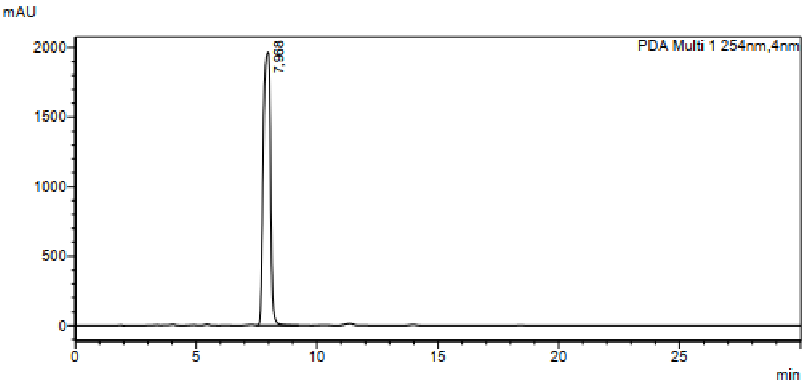

Peak Table

PDA Ch1 254nm

Peak#	Ret. Time	Area	Height	Conc.	Unit	Mark	Name
1	7.968	40736489	1965204	0.000		M	
Total		40736489	1965204				

*Chromatograph Shimadzu (pump: LC30AD Nexera X2, autosampler: SIL30AC Nexera X2, PDA detector: SPDM20A, thermostat: CTO20AC), mobile phase: MeOH/ H_2O/FA (formic acid) 50/50/0.1 (v/v/v). stationary phase: Kinetex C18 2.6 µm 150 x 4.6 mm, mobile phase flow: 0.8 mL / min, temp: 20°C, detection: PDA (channel 1: 254 nm + full spectrum in the range 190-800 nm).

Figure S20. HPLC data of **22***

2022-07-06 17:15:48 Page 1 / 1

SHIMADZU LabSolutions

Analysis Report

<Sample Information>

Sample Name	: 22W		
Sample ID	:		
Data Filename	: 22W.lcd		
Method Filename	: metoda.lcm		
Batch Filename	: batch.lcb		
Vial #	: 0-5	Sample Type	: Unknown
Injection Volume	: 10 uL		
Date Acquired	: 2022-07-06 16:04:27	Acquired by	: System Administrator
Date Processed	: 2022-07-06 17:10:30	Processed by	: System Administrator

<Chromatogram>

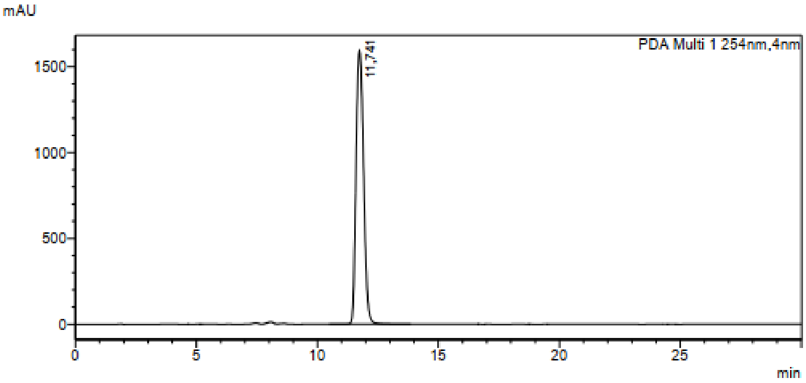

Peak Table

PDA Ch1 254nm

Peak#	Ret. Time	Area	Height	Conc.	Unit	Mark	Name
1	11.741	34738657	1592879	0.000			
Total		34738657	1592879				

*Chromatograph Shimadzu (pump: LC30AD Nexera X2, autosampler: SIL30AC Nexera X2, PDA detector: SPDM20A, thermostat: CTO20AC), mobile phase: MeOH/ H_2O/FA (formic acid) 50/50/0.1 (v/v/v). stationary phase: Kinetex C18 2.6 µm 150 x 4.6 mm, mobile phase flow: 0.8 mL / min, temp: 20°C, detection: PDA (channel 1: 254 nm + full spectrum in the range 190-800 nm).

Figure S21. Cell apoptosis of compounds **9-21** measured by flow cytometry using annexin V and PI. *Peripheral Blood Mononuclear Cells (PBMC)* were exposed to varying concentrations of test compounds (**9-20**). Untreated cells and cells exposed to DMSO (the highest percentage that are used together with compounds) were used as negative control. In each dot plot quadrant Q1: shows necrotic cells (Annexin V $^{-}$/ PI^{+}); Q2: late apoptotic cells (Annexin V^{+} /PI^{+}); Q3: early apoptotic cells (Annexin V^{+}/PI^{-}) and Q4: shows viable cells (Annexin V^{-} /PI^{-}).

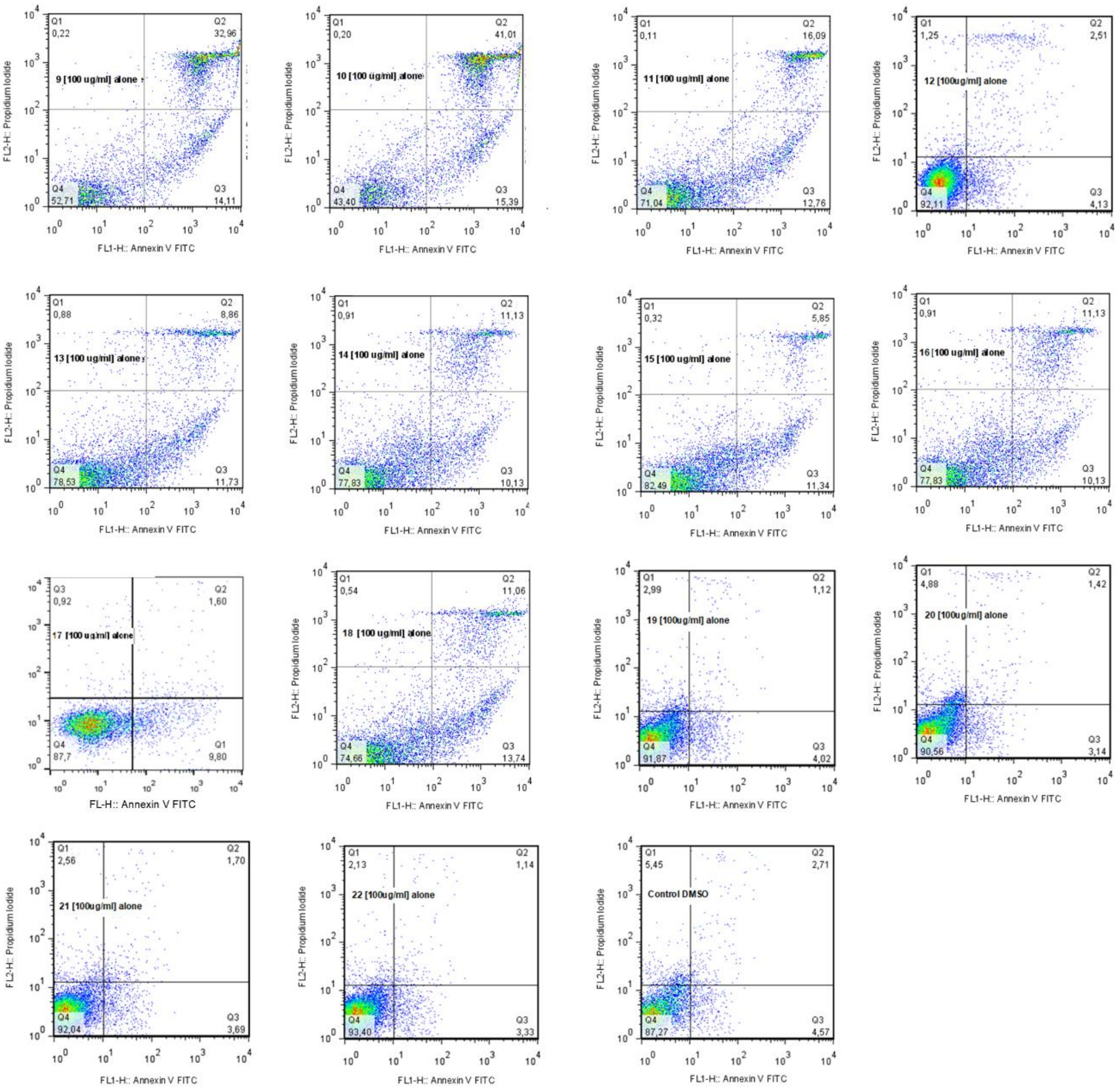

Supplement: Supplementary file 1 [file molecules-27-04488-s001.zip › molecules-1789665-supplementary.pdf]
